# Recent Progress on Graphitic Carbon Nitride–Based Photocatalysts for Hydrogen Peroxide Synthesis

**DOI:** 10.1002/EXP.20240484

**Published:** 2026-06-05

**Authors:** Liangpang Xu, Xixian Nan, Jimmy C. Yu

**Affiliations:** ^1^ Department of Chemistry The Chinese University of Hong Kong, Shatin, New Territories Hong Kong China

**Keywords:** artificial photosynthesis, catalyst modification, graphitic carbon nitride, hydrogen peroxide

## Abstract

Hydrogen peroxide (H_2_O_2_) is an indispensable chemical that is used in many industries. Artificial photosynthesis of H_2_O_2_ from oxygen and water has obtained worldwide attention due to the merits of low‐cost and sustainability. Graphitic carbon nitride (g‐C_3_N_4_)‐based semiconductors are one of the most popular photocatalysts for H_2_O_2_ synthesis due to the advanced intrinsic properties of g‐C_3_N_4_. To realize efficient H_2_O_2_ photosynthesis, multiple strategies have been proposed for boosting the photocatalytic activity of g‐C_3_N_4_. Herein, we summarize the rapid progress of H_2_O_2_ synthesis over g‐C_3_N_4_‐based photocatalysts in the last several years. The review begins with a brief introduction of H_2_O_2_ synthesis and g‐C_3_N_4_‐based photocatalysts, followed by the principle for photocatalytic H_2_O_2_ synthesis. Then, a series of common modification methods for preparing advanced g‐C_3_N_4_‐based photocatalysts are systematically introduced and discussed, including heteroatom doping, defect engineering, junction construction, metal cocatalyst loading, single atom loading, functionalization, and other strategies. Finally, the prospects and challenges for future development are discussed.

## Graphical abstract

This review summarizes recent progress in using graphitic carbon nitride (g‐C_3_N_4_) photocatalysts to produce hydrogen peroxide (H_2_O_2_). It highlights how solar energy can cleanly convert water and oxygen into this valuable chemical. We discuss key modification strategies, such as defect engineering, doping, and junction construction, to enhance efficiency while outlining paths for future industrial applications.

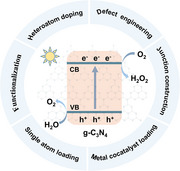

## 1 Introduction

As a versatile green oxidant, hydrogen peroxide (H_2_O_2_) is used in a variety of industries, including chemical synthesis [1], environmental remediation [2], disinfection [3], and one‐compartment fuel cells [4]. It is one of the top 100 chemicals in the world with an annual production of 5.5 million tons. To date, the anthraquinone (AQ) oxidation process remains the predominant industrial method for synthesizing more than 95% of the total production of H_2_O_2_ [5]. However, the AQ method suffers from severe energy and environmental issues, including the extensive energy consumption in the hydrogenation and oxidation processes, as well as the generation of a large amount of wastewater, exhaust gas, and solid waste [5, 6].

Thus, production of H_2_O_2_ in a more sustainable approach has triggered increasing research interests, such as electrochemical and photocatalytic methods [7, 8]. Semiconductor‐based photosynthesis of H_2_O_2_ using the ubiquitous O_2_ and water is a promising route in this context. Under solar irradiation, a proper photocatalyst could generate free electrons to induce the O_2_ reduction reaction (ORR) with H_2_O_2_ as the product [8]. Such an ideal system only requires input of free solar energy and would not lead to environmental pollution. The H_2_O_2_ yield strongly depends on the photocatalytic activity of semiconductors, thus giving rise to intensive studies on the development of various advanced photocatalysts. Metal‐based materials, such as TiO_2_, ZnO, BiVO_4_, CdS, and their derived materials, have long been the research topic [8, 9, 10, 11]. Nevertheless, most of them suffer from such limitations as low activity, difficult modification of structure and interface properties, metal leaching to cause environmental pollution, and high cost due to usual noble metal loading [12]. By contrast, metal‐free semiconductors, usually polymeric conjugated polymers, could be considered more attractive photocatalysts by avoiding these issues. The classic graphitic carbon nitride (g‐C_3_N_4_) has been extensively used in this context [8, 13]. More recently, covalent organic frameworks, a class of porous organic polymers with crystalline and periodic structures, have also emerged for efficient ORR photocatalysis because of the flexible molecular structure design and exciton dynamics modulation [14, 15]. These polymer photocatalysts demonstrate unique electronic and optical properties with suitable band structures for sunlight absorption, enabling diverse photocatalytic reactions, including ORR [12, 16]. Their designable, adjustable, and functionalizable nature further offers opportunities to enhance catalytic activity and stability [17, 18].

g‐C_3_N_4_ is a class of polymeric material composed primarily of carbon and nitrogen. It has a history dating back to the early 19th century. The first reported carbon nitride, paracyanogen, was discovered by Berzelius in 1834. However, significant interest in g‐C_3_N_4_ emerged in the late 20th century due to its promising electronic, catalytic, and mechanical properties. g‐C_3_N_4_ was first used for photocatalysis by Wang et al. in 2009 for visible‐light photocatalytic water splitting [19]. Since then, g‐C_3_N_4_ has become a brilliant new star in photocatalysis, which has triggered intensive research interests in a series of other fields such as H_2_ evolution [20], environmental treatment [21], and CO_2_ reduction [22, 23]. Based on its allotropes, g‐C_3_N_4_ falls into categories such as traditional triazine/heptazine melon and emerging structures like poly(heptazine imide) (PHI) and poly(triazine imide) – the latter attracting significant interest for superior photocatalytic properties [24, 25, 26, 27]. The significant advantages of g‐C_3_N_4_ photocatalysts include the following: [28, 29] (1) the band gap of ∼2.7 eV allows visible‐light utilization; (2) the electronic bands cover the redox potentials of classic reactions such as water oxidation and O_2_ reduction; (3) the specific microstructure provides abundant active sites, including the surface termination as defects and nitrogen atoms for electron localization or anchoring inorganic/organic functional motifs; and (4) the chemical and thermal stability and anti‐photocorrosion property guarantee its reusability in photocatalytic reactions. Therefore, g‐C_3_N_4_ has become an emerging photocatalyst with great potential to boost the artificial photosynthesis of H_2_O_2_.

Fabrication of g‐C_3_N_4_‐based photocatalysts with high H_2_O_2_ synthesis activity is the mainstream research direction due to the poor efficiency of bare g‐C_3_N_4_. The targets of catalyst modification are centered on accelerating the separation of charge carriers, improving the visible‐light absorption, and creating rich active sites for O_2_ adsorption and reduction [10, 30]. To date, a series of modifications of g‐C_3_N_4_ have been proposed to elevate the photocatalytic performance on H_2_O_2_ production, including heteroatom doping [31], defect engineering [32], junction construction [33], single atom (SA) loading [34], functionalization, and other strategies [35, 36]. For example, the construction of Z‐scheme Bi_4_O_5_Br_2_/g‐C_3_N_4_ heterojunctions promotes H_2_O_2_ yield by raising the charge separation efficiency [37]; the transformation of terminal amino group into cyano groups can adjust the band structure of g‐C_3_N_4_ and provide sites for oxygen adsorption and protonation to achieve enhanced activity [38]; and the coordination of Ni SAs onto g‐C_3_N_4_ provides advanced catalytically active sites for O_2_ reduction to produce H_2_O_2_, giving rise to an apparent quantum yield (Φ_AQY_) of 10.9% at 420 nm and a solar‐to‐chemical conversion efficiency of 0.82% [34].

In view of the rapid expansion of g‐C_3_N_4_‐based materials for H_2_O_2_ photosynthesis, it's quite necessary to summarize the most recent progress, hopefully providing guidance for future catalyst design toward efficient H_2_O_2_ production. This review begins with the introduction of H_2_O_2_ synthesis and g‐C_3_N_4_ photocatalysts, followed by the principle of photocatalytic H_2_O_2_ generation. Then, the dominant part comes to a systematic discussion of the progress in g‐C_3_N_4_‐based photocatalysts for H_2_O_2_ synthesis, which is divided by different types of modification on pristine g‐C_3_N_4_. In the end, we conclude the progress and prospects of the remaining challenges and future advances for g‐C_3_N_4_‐based photosynthesis of H_2_O_2_.

## 2 Principle for Photocatalytic H_2_O_2_ Synthesis

**FIGURE 1:**
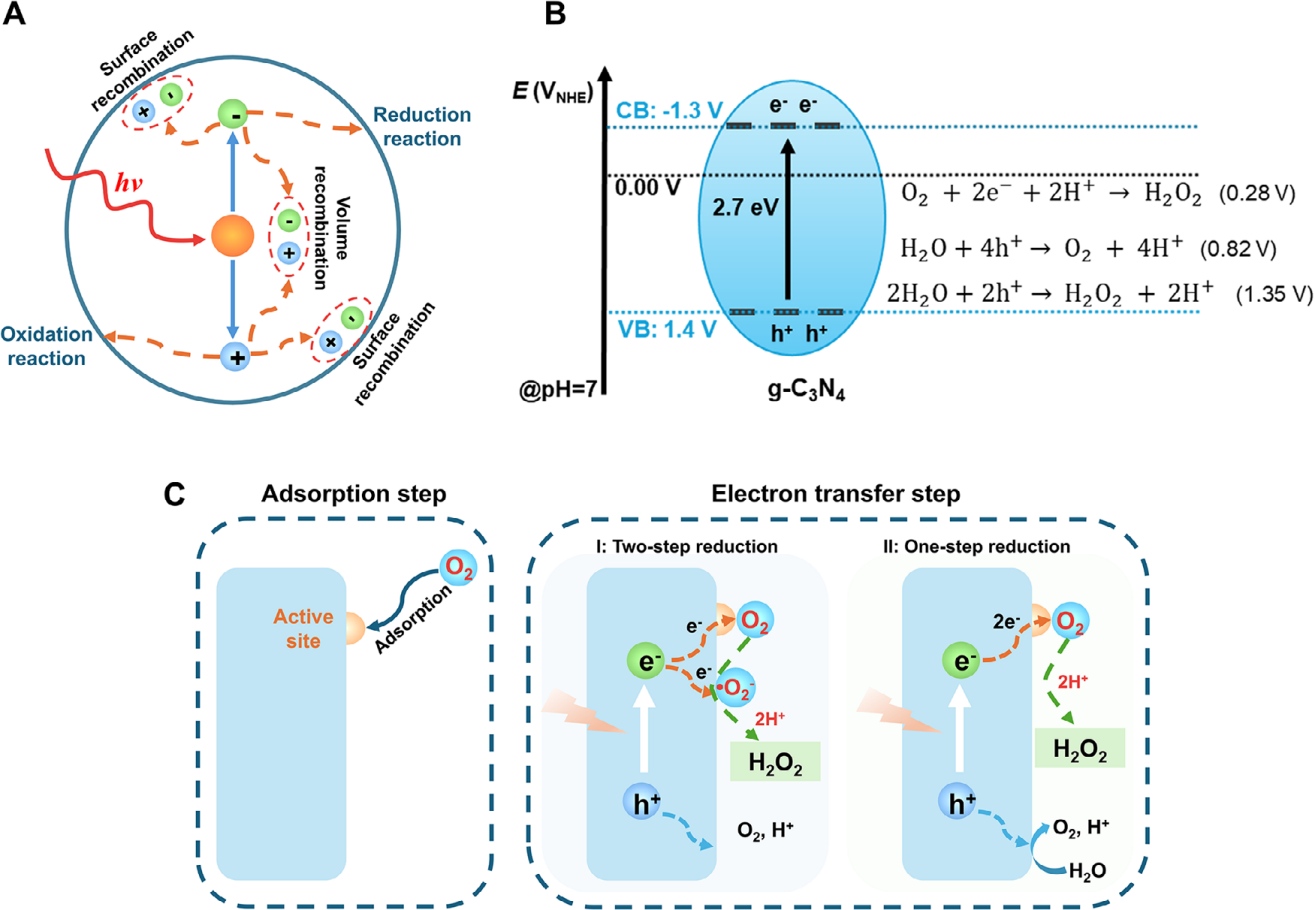
(A) Photoexcitation and charge‐decay pathway in a photocatalyst. (B) Energy diagrams of g‐C_3_N_4_ and standard redox potentials for H_2_O_2_ production. (C) Schematic illustration of the process of photocatalytic production of H_2_O_2_.

In a typical photocatalytic process, as shown in Figure 1A, free electrons (e^−^) are generated once the semiconductor gets excited by irradiation of light with photon energy higher than the band gap. With the excitation of e^−^ from the valence band (VB) to the conduction band (CB), positively charged holes (h^+^) are thereby left in the VB. Then, the photo‐induced e^−^ transfer to the surface of the catalyst induces reduction reactions, while the h^+^ inversely leads to oxidation reactions [39]. Meanwhile, volume and surface recombination of photo‐induced e^−^ and h^+^ would also occur before they are involved in redox reactions.

Photocatalytic production of H_2_O_2_ is generally from the ORR with the participation of H^+^ from water (Equations (1)–(5)). The other potential pathway of water oxidation reaction (WOR) to generate H_2_O_2_, however, is unfavorable due to the uphill thermodynamics (Figure 1B, Equations (6) and (7)) [40]. That is, the H_2_O_2_ is an unstable product and would be immediately decomposed into O_2_ and water at such a high potential (1.35 V vs. NHE). The pathway of ORR for H_2_O_2_ generation firstly requires active sites on the surface of catalysts for the adsorption of O_2_ molecules (Figure 1C). Then, once the photo‐induced e^−^ transfer to the active site, it could be captured by O_2_ molecules, thus leading to the reduction of O_2_.

The O_2_ reduction to form H_2_O_2_ could be achieved by a two‐step or, directly, a one‐step reduction process (Figure 1C, Equations (1)–(5)) [41]. Equations (1)–(4) show the H_2_O_2_ generation via the sequential two‐step single‐electron reduction pathway. Above all, the O_2_ molecule obtains one e^−^ from the catalyst to form the superoxide anion radical (•O_2_^−^) (Equation (1)), which then transforms into •HO_2_^−^ by reacting with H^+^ (Equation (2)). This •HO_2_^−^ intermediate is further readily reduced by an e^−^ (Equation (3)), and the resulting HO_2_^−^ anion obtains a H^+^ to finally generate H_2_O_2_ (Equation (4)). In addition, the O_2_ reduction can also proceed in a two‐electron pathway, realizing H_2_O_2_ generation by a direct one‐step reduction process. The O_2_ receives 2e^−^ from the catalyst to form O_2_^2−^, and its binding with two H^+^ induces direct H_2_O_2_ formation (Equation (5)). On the other hand, the h^+^ in the VB is typically consumed in WOR to generate O_2_ in a pure water system (Equation (7)).

(1) $$\begin{array}{cc} O_{2}+e^{-}\;\to {\;•O}_{2}^{-} & \left(-0.33\;V\;\mathrm{vs}.\;\mathrm{NHE}\right) \end{array}$$

(2) $${•O}_{2}^{-}+H^{+}\;\to \;{•\mathrm{HO}}_{2}^{-}\;$$

(3) $$\begin{array}{cc} \;•{\mathrm{HO}}_{2}^{-}+e^{-}\;\to \;{\mathrm{HO}}_{2}^{-} & \left(1.44\;V\;\mathrm{vs}.\;\mathrm{NHE}\right) \end{array}$$

(4) $${\mathrm{HO}}_{2}^{-}+H^{+}\;\to \;H_{2}O_{2}\;$$

(5) $$\begin{array}{cc} O_{2}+2e^{-}+2H^{+}\;\to \;H_{2}O_{2} & \left(0.69\;V\;\mathrm{vs}.\;\mathrm{NHE}\right) \end{array}$$

(6) $$\begin{array}{cc} 2H_{2}O+2h^{+}\;\to \;H_{2}O_{2}+2H^{+} & \left(1.35\;V\;\mathrm{vs}.\;\mathrm{NHE}\right) \end{array}$$

(7) $$\begin{array}{cc} H_{2}O+4h^{+}\;\to \;O_{2}+4H^{+} & \left(0.82\;V\;\mathrm{vs}.\;\mathrm{NHE}\right) \end{array}$$

Distinguishing between the one‐electron and two‐electron ORR pathways for H_2_O_2_ production on g‐C_3_N_4_ is critical. The most definitive method employs a rotating ring‐disk electrode to determine the electron transfer number (*n*): *n*≈1 signifies a one‐electron pathway (forming •O_2_^−^), whereas *n*≈2 indicates a direct two‐electron route [41]. Electron paramagnetic resonance (EPR) spectroscopy can detect •O_2_^−^ intermediates, supporting the one‐electron mechanism [16]. Theoretical calculations provide complementary evidence by comparing the free energy barriers for *OOH formation versus direct H_2_O_2_ generation; a lower barrier for *OOH favors the two‐electron path [42]. A combination of these techniques enables a conclusive distinction.

A photocatalyst should meet various requirements for realizing efficient synthesis of H_2_O_2_. Thermodynamically, the redox capacities of photo‐induced e^−^ and h^+^ should be strong enough to drive ORR and WOR, respectively (Figure 1B). Specifically, the CB potential of photocatalysts should be below −0.33 V and 0.69 V for two‐step and one‐step pathways (Equations (1)–(5)), respectively. The VB potential needs to be larger than 0.82 V (vs. NHE) for WOR to produce O_2_ (Equation (7)). Moreover, given the common overpotential for redox reactions, the requirements for CB and VB potentials would be harsher. For the band gap of photocatalysts, it needs to be over 1.56 eV to ensure the bilateral reactions. In terms of kinetics, it dominantly depends on the e^−^ supply and the amount of intrinsic active sites of the catalysts. The e^−^ supply is usually favored by a stronger light absorption, a higher charge separation efficiency, a faster charge transfer rate, etc. The amount of active sites depends on the surface areas and the crystal defects. Besides, as a reaction involving gas, the O_2_ diffusion in aqueous solution suffers from a significant limitation, thus inspiring the reaction system design, such as the construction of a gas–liquid–solid three‐phase interface to enhance the mass transport for efficient reaction kinetics [43, 44]. The reaction conditions, such as pH and temperature, also closely affect the H_2_O_2_ production kinetics.

## 3 Recent g‑C_3_N_4_‐Based Photocatalysts for H_2_O_2_ Synthesis

**FIGURE 2:**
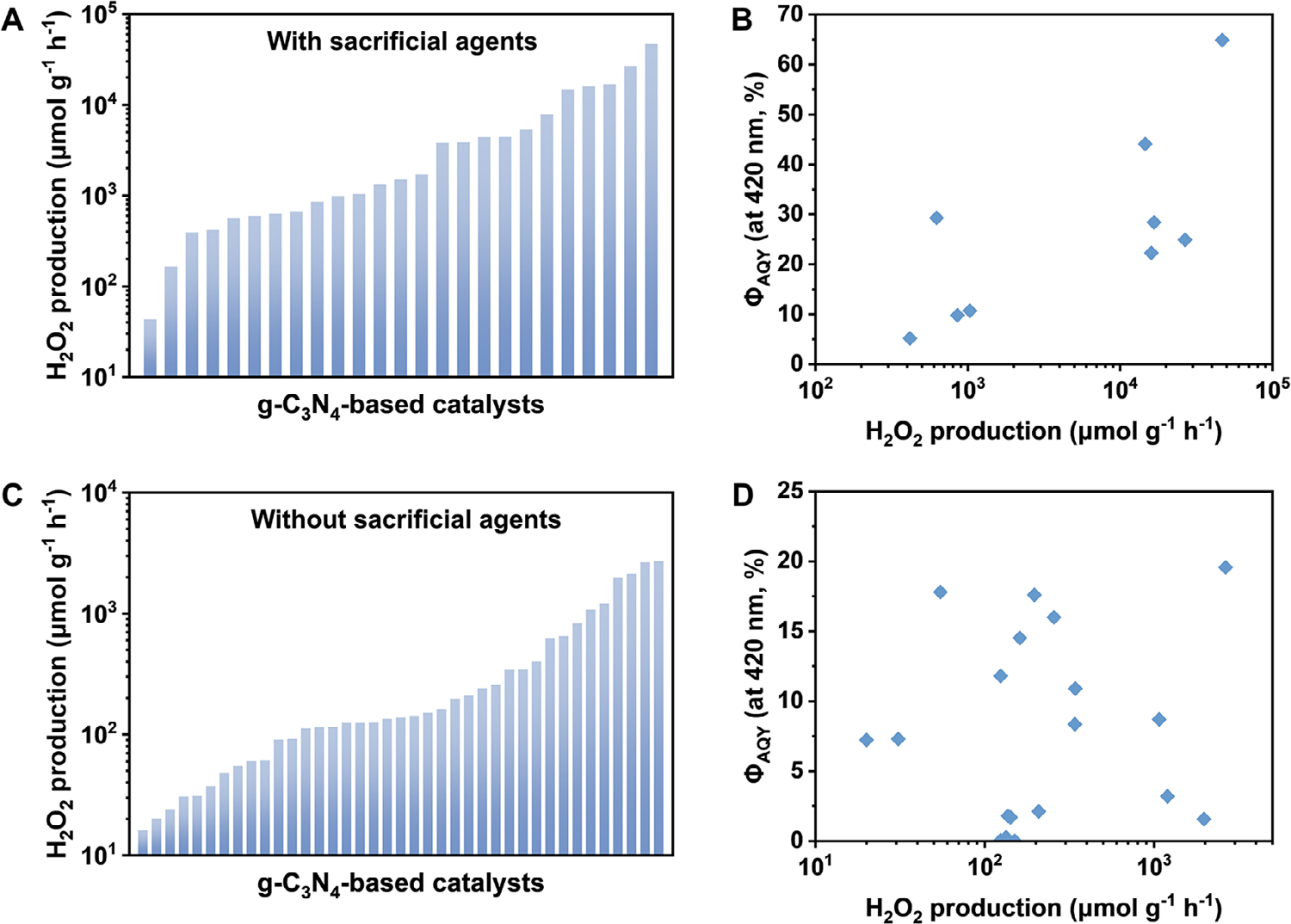
Performance summary of g‐C_3_N_4_‐based photocatalysts on H_2_O_2_ synthesis. (A) and (C) Statistical distribution of reported H_2_O_2_ production rates with or without sacrificial agents. (B) and (D) The Φ_AQY_ coupled with corresponding H_2_O_2_ generation rates with or without sacrificial agents.

Despite the great interest in g‑C_3_N_4_‐based materials for H_2_O_2_ photosynthesis, the catalytic activity is still far from practical applications. The restrictions mainly include rapid recombination of the photoinduced charge carriers and a lack of active sites for O_2_ adsorption and activation. To address these issues, various advanced approaches such as doping heteroatoms, engineering defects, construction of heterojunctions, loading metal SAs, and functionalization have been continuously adopted to boost the activity of g‑C_3_N_4_ for H_2_O_2_ photosynthesis (Figure 2 and Tables 1 and 2). Herein, we present the recent development of these methods and their principles for promoting H_2_O_2_ photosynthesis over g‐C_3_N_4_.

**TABLE 1:** Summary of the recent g‐C_3_N_4_‐based photocatalysts for the production of H_2_O_2_ using sacrificial agents.

Photocatalysts	Sacrificial agent	Irradiation	Yield (µmol g^−1^ h^−1^)	Φ_AQY_ (%)	Ref.
KCN	10% IPA	300 W Xe lamp (400−780 nm)	4400	NT	[45]
MPCN	10% IPA	300 W Xe lamp (λ ≥ 420 nm)	4424	NT	[46]
3DPCN	10% IPA	300 W Xe lamp (λ ≥ 420 nm)	1321	NT	[47]
IO CN‐Cv	5% EtOH	300 W Xe lamp (λ ≥ 420 nm)	162.9	NT	[48]
O‐CNC	10% IPA	300 W Xe lamp (λ ≥ 420 nm)	389.2	NT	[49]
Cv‐PCNNS	10% IPA	300 W Xe lamp (λ ≥ 420 nm)	984.8	NT	[50]
UCNS	20% EtOH	300 W Xe lamp (λ ≥ 420 nm)	416.7	5.19	[51]
Nv‐M	5% IPA	300 W Xe lamp (λ ≥ 420 nm)	623.5	29.3	[52]
DDCN	10% MeOH	300 W Xe lamp (λ ≥ 420 nm)	1031	10.7	[53]
W_18_O_49_/g‐C_3_N_4_	10% IPA	full spectrum (100 mW cm^−2^)	555	NT	[54]
CN‐NH_4_‐NaK	10% IPA	300 W Xe lamp (λ ≥ 420 nm)	16675	28.4	[55]
MgIn_2_S_4_/g‐C_3_N_4_	10% EtOH	250 W visible light	1700	NT	[56]
ZnIn_2_S_4_/g‐C_3_N_4_	10% IPA	AM 1.5G	854.68	9.82	[57]
C_3_N_4_/PDA	10% EtOH	300 W Xe lamp (λ ≥ 350 nm)	3801.3	NT	[58]
ZnO/g‐C_3_N_4_	10% EtOH	500 W Xe arc lamp (320−780 nm)	664.1	NT	[59]
Zn‐TCPP/CN	10% EtOH	300 W Xe lamp	591.9	NT	[60]
ZCN12	10% EtOH	300 W Xe lamp (λ ≥ 350 nm)	3860	NT	[61]
Sb/Cv‐C_3_N_4_	10% IPA	300 W Xe lamp	5369	11.66 (350 nm)	[62]
m‐CNNP	10% IPA	300 W Xe lamp (λ ≥ 420 nm)	43.07	1.0	[36]
Na‐PCN	10% EtOH	300 W Xe lamp (λ > 400 nm)	16,010	22.3	[63]
Au/CN‐100	5% IPA	300 W Xe lamp (λ > 400 nm)	1502	NT	[64]
CNK	10% EtOH	Xe lamp (λ ≥ 420 nm)	46,800	64.9	[65]
ATC‐CNK	5% EtOH	solar simulator	26,700	24.9	[66]
C_3_N_4_−Zn−N(C)	10% EtOH	300 W Xe lamp (λ ≥ 365 nm)	7800	26.8 (365 nm)	[67]
A‐CN	100 mM FFA	300 W Xe lamp (λ ≥ 420 nm)	14,600	44.1	[68]

*Note*: Φ_AQY_ is at 420 nm if not specified.

Abbreviations: NT: not tested; IPA: isopropanol; MeOH: methanol; EtOH: ethanol; FFA: furfuryl alcohol.

**TABLE 2:** Summary of the recent g‐C_3_N_4_‐based photocatalysts for the production of H_2_O_2_ without using sacrificial agents.

Photocatalysts	Irradiation	Yield (µmol g^−1^ h^−1^)	Φ_AQY_ (%)	Ref.
g‐CN‐MI‐40	300 W Xe arc lamp (λ>420 nm)	∼20	7.23	[31]
KCNOH‐5	300 W Xe lamp (λ ≥ 420 nm)	91.36	NT	[69]
CPN	Visible light (420−700 nm)	1968	1.57	[70]
PCN	300 W Xe lamp (λ ≥ 420 nm)	1071.7	8.70	[71]
Cv‐g‐C_3_N_4_	300 W Xe lamp (λ ≥ 420 nm)	90	NT	[72]
5Cv@g‐C_3_N_4_	300 W Xe lamp (λ ≥ 420 nm)	124.5	NT	[38]
BNCN	300 W Xe lamp (λ ≥ 420 nm, 202.24 mW cm^−2^)	160.9	14.52	[73]
Nv‐C≡N‐CN	300 W Xe lamp (λ ≥ 420 nm, 40 mW cm^−2^)	137	1.8	[74]
CHF‐DPDA	300 W Xe lamp (λ ≥ 420 nm)	256	16	[75]
ZnPPC‐NBCN	300 W Xe lamp (400−800 nm)	114	NT	[76]
PI5.0‐NCN	300 W Xe lamp (λ ≥ 420 nm)	1200	3.2	[77]
Cu_2_(OH)PO_4_/g‐C_3_N_4_	300 W Xe lamp	∼2700	NT	[78]
g‐C_3_N_4_/PDI‐BN‐rGO	300 W Xe lamp (λ ≥ 420 nm)	30.8	7.3	[79]
AgBr‐Br‐g‐C_3_N_4_	250 W high‐pressure Na lamp (λ = 400 ∼ 800 nm)	650	NT	[80]
Bi_4_O_5_Br_2_/g‐C_3_N_4_	300 W Xe lamp (λ ≥ 420 nm)	124	11.8	[37]
α‐Fe_2_O_3_/ CQD@g‐C_3_N_4_	300 W Xe lamp (λ ≥ 420 nm)	54.6	17.8	[81]
RF‐CN‐bm	300 W Xe lamp (λ ≥ 420 nm)	16	NT	[82]
HJ‐C_3_N_4_	300 W Xe lamp (λ ≥ 420 nm)	115	NT	[83]
g‐C_3_N_4_/Co_9_S_8_	300 W Xe lamp	826	18.1 (450 nm)	[84]
SCN/T9	300 W Xe lamp (300−700 nm)	2128	0.61 (365 nm)	[85]
g‐C_3_N_4_/α‐MnS	300 W Xe lamp	111.6	8.5 (450 nm)	[86]
ORP‐g‐C_3_N_4_	300 W Xe lamp (λ ≥ 420 nm)	48	NT	[87]
CN/rGO@BPQDs	300 W Xe lamp (λ ≥ 420 nm)	60.6	NT	[88]
MMO@C_3_N_4_	300 W Xe lamp full spectrum	∼60	NT	[89]
ZIF‐8/C_3_N_4_	300 W Xe lamp (λ ≥ 420 nm)	2641	19.57	[90]
NiSAPs‐PuCN	300 W Xe lamp (λ ≥ 420 nm)	342.2	10.9	[91]
Co/AQ/C_3_N_4_	AM 1.5G	124	0.054	[92]
Sb‐SACS	300 W Xe lamp (λ ≥ 420 nm)	196	17.6	[93]
g‐C_3_N_4_/PEI	arc Xe lamp (AM 1.5)	208.1	2.12	[94]
g‐C_3_N_4_‐NH‐CH_2_‐OH	5 W LED lamp (λ > 420 nm)	30.4	NT	[35]
g‐C_3_N_4_/PIM‐1	300 W Xe lamp (λ ≥ 420 nm)	37	NT	[95]
P,N‐C@CNHS	300 W Xe lamp (λ ≥ 420 nm)	239.5	NT	[96]
CM‐CN_3_	300 W Xe lamp (λ ≥ 420 nm)	150	2.2%	[97]
UPCN	300 W Xe lamp (λ ≥ 420 nm)	23.91	NT	[98]
TA‐CN‐3	300 W Xe lamp (λ ≥ 420 nm)	142	1.7	[99]
Na‐PCN	300 W Xe lamp (λ > 400 nm)	133.48	0.26	[63]
Pt‐NaCN	300 W Xe lamp (400−800 nm)	∼400	NT	[100]
PT‐KCN	300 W Xe lamp (λ ≥ 420 nm)	620	NT	[101]
CoO*_x_*‐BCN‐FeOOH	300 W Xe lamp (λ ≥ 420 nm)	340	8.36	[102]

*Note*: Φ_AQY_ is at 420 nm if not specified.

Abbreviation: NT: not tested.

### 3.1 Heteroatom Doping

Doping additional elements and impurities into the g‐C_3_N_4_ is a critical strategy for improving the photocatalytic activity. Heteroatom doping can effectively tune the optical, electronic, and other physical properties of g‐C_3_N_4_, thus leading to extension of light absorption and efficient separation of charge carriers [103, 104]. For light absorption, non‐metal dopants such as P and S narrow the bandgap (e.g., from 2.74 to 2.57 eV) by forming new hybridized states (P–N, S–C), extending absorption edges beyond 550 nm [105]. Metal dopants (e.g., Fe, Cu) introduce intermediate energy levels, enabling sub‐bandgap photon utilization. Concurrently, dopants can optimize charge dynamics – polar atoms (B, O) create built‐in electric fields to drive charge separation, while transition metals (Co, Ni) act as electron traps via d‐orbitals, suppressing recombination. To date, studies focusing on doping of g‐C_3_N_4_ have been extensively reported [106], and a number of dopants have been successfully introduced to g‐C_3_N_4_ for enhanced photocatalytic H_2_O_2_ synthesis. These dopants include nonmetal elements like O, S, P, as well as metals such as alkaline metals (e.g., Li^+^, Na^+^, K^+^) and transition metals (e.g., Fe, Cu) [107].

#### 3.1.1 Non‐Metal Doping

**FIGURE 3:**
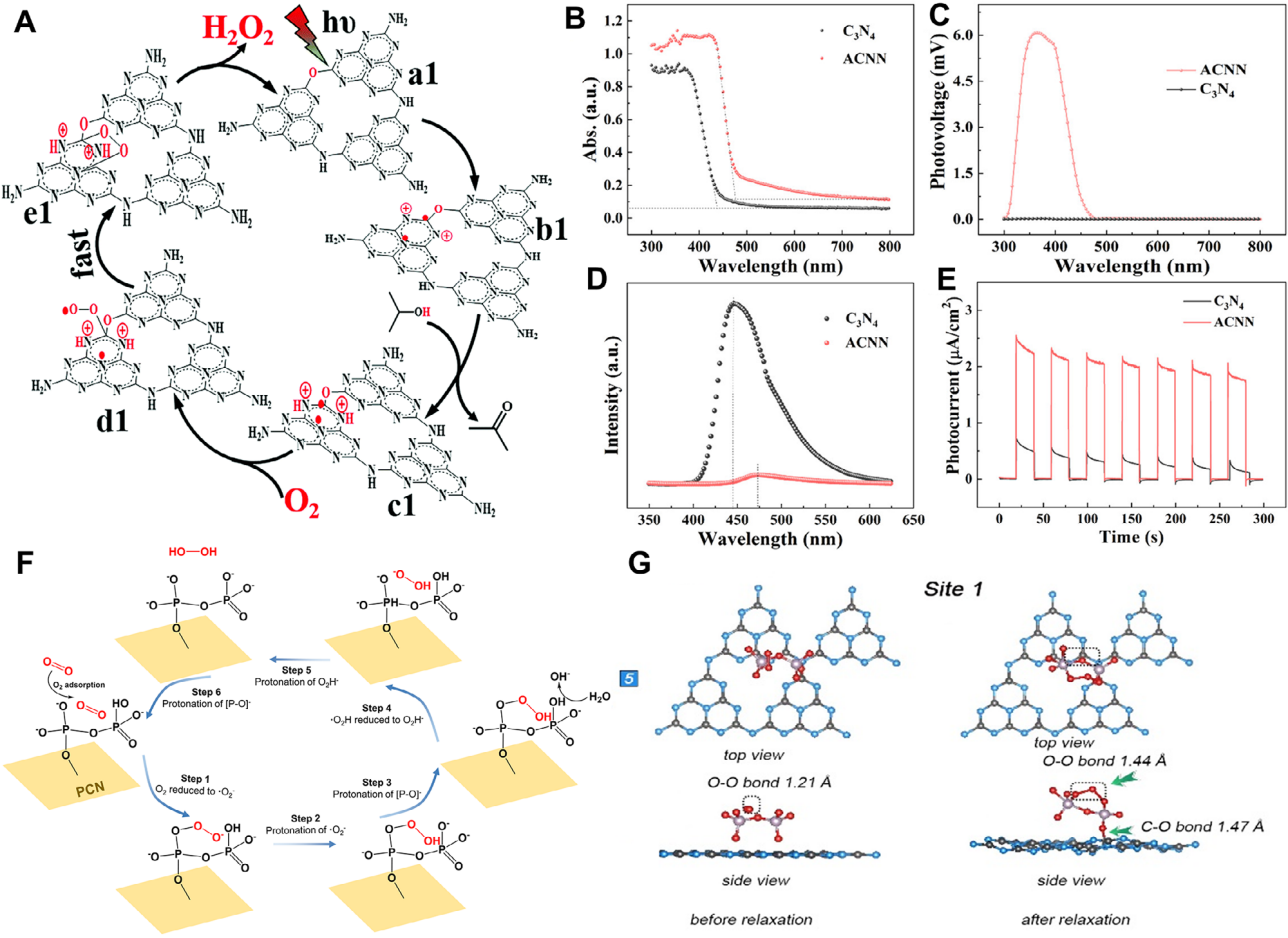
(A) The mechanism of oxygen‐doped g‐C_3_N_4_ for ORR. Reproduced with permission [42]. Copyright 2018, Royal Society of Chemistry. (B)–(E) UV–vis diffuse reflection spectra (DRS), surface photovoltage (SPV), photoluminescence (PL), and photocurrent–time curves of alkali metal‐doped g‐C_3_N_4_ with N vacancies (ACNN) and pristine g‐C_3_N_4_ (C_3_N_4_). Reproduced with permission [108]. Copyright 2020, American Chemical Society. (F) Schematic illustration of alkaline H_2_O_2_ generation process on pyrophosphate‐incorporated g‐C_3_N_4_ (PCN). (G) Adsorption and activation process of O_2_ on g‐C_3_N_4_ and PCN. Reproduced with permission [71]. Copyright 2023, Elsevier.

The C/N atoms in g‐C_3_N_4_ can be easily substituted by non‐metal elements such as O and P. Wei et al. doped O into two‐dimensional (2D) g‐C_3_N_4_ (denoted as OCN) to promote the charge transfer and two‐electron O_2_ reduction pathway [42]. This photocatalyst shows an AQY of 10.2% at 420 nm for H_2_O_2_ production, 3.5 times higher than that of g‐C_3_N_4_. It was found that the ORR on OCN preferentially produces 1,4‐endoperoxide species rather than superoxide radicals, thus giving rise to a high selectivity for H_2_O_2_ generation (Figure 3A). Moreover, OCN exhibits higher oxygen reducibility and charge separation efficiency than g‐C_3_N_4_. In another work, Iqbal and co‐workers designed 3D hierarchical P‐doped, interconnected, flower‐like macroporous g‐C_3_N_4_ [47]. It was revealed that the P‐doped g‐C_3_N_4_ exhibits enhanced electronic interactions and improved charge separation efficiency, and the interconnected P‐doped porous framework accelerates the chemical reaction on the surface of the photocatalyst. As a result, a 7.2‐fold enhancement on H_2_O_2_ production (1321.3 µmol g^−1^ h^−1^) was obtained.

#### 3.1.2 Metal Doping

Metal dopants, especially alkaline metals (e.g., Li^+^, Na^+^, K^+^), are known for promoting the photocatalytic activity of g‐C_3_N_4_ by facilitating light absorption, charge separation, and transfer. Wu et al. introduced alkali metal dopants (Na^+^ and K^+^) and N vacancies (N_V_) on g‐C_3_N_4_ (ACNN) to simultaneously enhance the light absorption and promote the charge separation [108]. UV–vis diffuse reflection spectra (DRS) demonstrated the broadened light absorption edge and stronger light absorbance of ACNN relative to g‐C_3_N_4_ (Figure 3B). The surface photovoltage (SPV), photoluminescence (PL), and photocurrent–time curves confirm the enhanced separation and inhibited recombination of charge carriers for ACNN (Figure 3C–E). Accordingly, the photocatalytic H_2_O_2_ production rate for ACNN reaches 10.2 mmol g^−1^ h^−1^, up to 89.5 times that of pristine g‐C_3_N_4_. Zhang et al. prepared K^+^‐intercalated porous crystalline g‐C_3_N_4_ with cyano group modification (KCN) [45]. Given the multiple optimizations, including the incorporation of K^+^, high crystallinity, and the cyano group, both the activity and selectivity of the catalyst on H_2_O_2_ production were significantly enhanced. The H_2_O_2_ production rate for KCN (11.2 mmol g^−1^ h^−1^) reaches approximately 17 times higher than that of pristine g‐C_3_N_4_. It was found that the facilitated adsorption and transfer of O_2_ and H^+^ accelerate surface reaction kinetics, and the rotating disk electrode measurements demonstrated the higher selectivity of KCN for the two‐electron O_2_ reduction pathway, while 4e^−^ O_2_ reduction occurs over pristine g‐C_3_N_4_.

Transition metals can also be introduced into g‐C_3_N_4_ for improving H_2_O_2_ synthesis efficiency, typically by serving as active sites. Hu et al. prepared hollow Cu‐doped g‐C_3_N_4_ with Cu^+^ inserted at the interstitial position via Cu(I)‐N bond coordination [109]. The Cu(I)‐N can not only serve as a favorable active site to adsorb and activate O_2_ molecules but also bridge the electron transfer from the catalyst to the adsorbed O_2_. As a result, both the photocatalytic activity and stability of the Cu‐doped g‐C_3_N_4_ for H_2_O_2_ production have been optimized. Similarly, Zhu et al. reported Fe‐doped g‐C_3_N_4_ nanosheets with a loading of ionic liquids [Bmim][Tf_2_N] [110]. The Fe doping together with the ionic liquid coating improves the photocatalytic H_2_O_2_ production rate by 25.9 fold compared with pristine g‐C_3_N_4_. Moreover, taking advantage of efficient H_2_O_2_ generation, Fe^3+^ on g‐C_3_N_4_ can further facilitate the Fenton reaction, thus giving rise to rapid organic pollutant degradation.

#### 3.1.3 Multi‐Element Doping

More recently, it has been more popular to introduce multiple elemental dopants onto g‐C_3_N_4_ for realizing synergetic enhancement on photocatalytic activity. Xu et al. incorporate pyrophosphate (P_2_O_7_^4−^) into g‐C_3_N_4_ (PCN) photocatalyst to address the issue of lack of protons for H_2_O_2_ generation in alkaline conditions [71]. The PCN is efficient for alkaline H_2_O_2_ synthesis with a rate of 1071.7 µmol g^−1^ h^−1^ (at pH 9) and an AQY of 8.7 % (at 420 nm). They demonstrated the roles of pyrophosphate as a proton shuttle to effectively adsorb and provide protons (Figure 3F), as well as advanced active sites for adsorption and activation of O_2_ – the adsorption energy of O_2_ is quite negative (−4.11 eV), and the O═O bond elongates obviously after adsorption (Figure 3G). Yuan et al. developed P, K‐codoped and crystalline g‐C_3_N_4_ (denoted as MPCN) with cyano groups [46]. The H_2_O_2_ synthesis rate for this photocatalyst (4424 µmol g^−1^ h^−1^) is up to 81 times higher than that of the pristine g‐C_3_N_4_. Mechanism study reveals that the terminal cyano groups enhance visible light absorption, while the K^+^ intercalation narrows the band gap and favors the charge carriers’ migration in adjacent layers, and the P–N bond and more polymerized crystalline structure greatly accelerate in‐plane charge transfer.

### 3.2 Defect Engineering

Defective g‐C_3_N_4_ has been widely developed due to its excellent optoelectronic and catalytic properties [111]. Creating defects can alter the electronic band structures by inducing mid‐gap states within the VB and CB, leading to enhanced light absorption and red shift of the absorption edge of semiconductors. The vacancies either create mid‐gap states below the CB to enable sub‐bandgap photon absorption or induce localized electron states near the VB to narrow the effective bandgap. The vacancies also enhance π‐electron delocalization in the tri‐s‐triazine units, strengthening visible‐light absorption intensity. Furthermore, the photo‐generated e^−^ in CB would transfer to the vacancy sites and get trapped instead of directly recombining with h^+^ in VB, which could largely delay the charge recombination process. More importantly, the defect of vacancies as electron‐rich centers has been recognized as effective adsorption sites or reactive sites to enhance various photocatalytic reactions. Given these advantages, many studies have demonstrated the critical role of defects on g‐C_3_N_4_ photocatalysts for improving the activity for the production of H_2_O_2_ [32].

**FIGURE 4:**
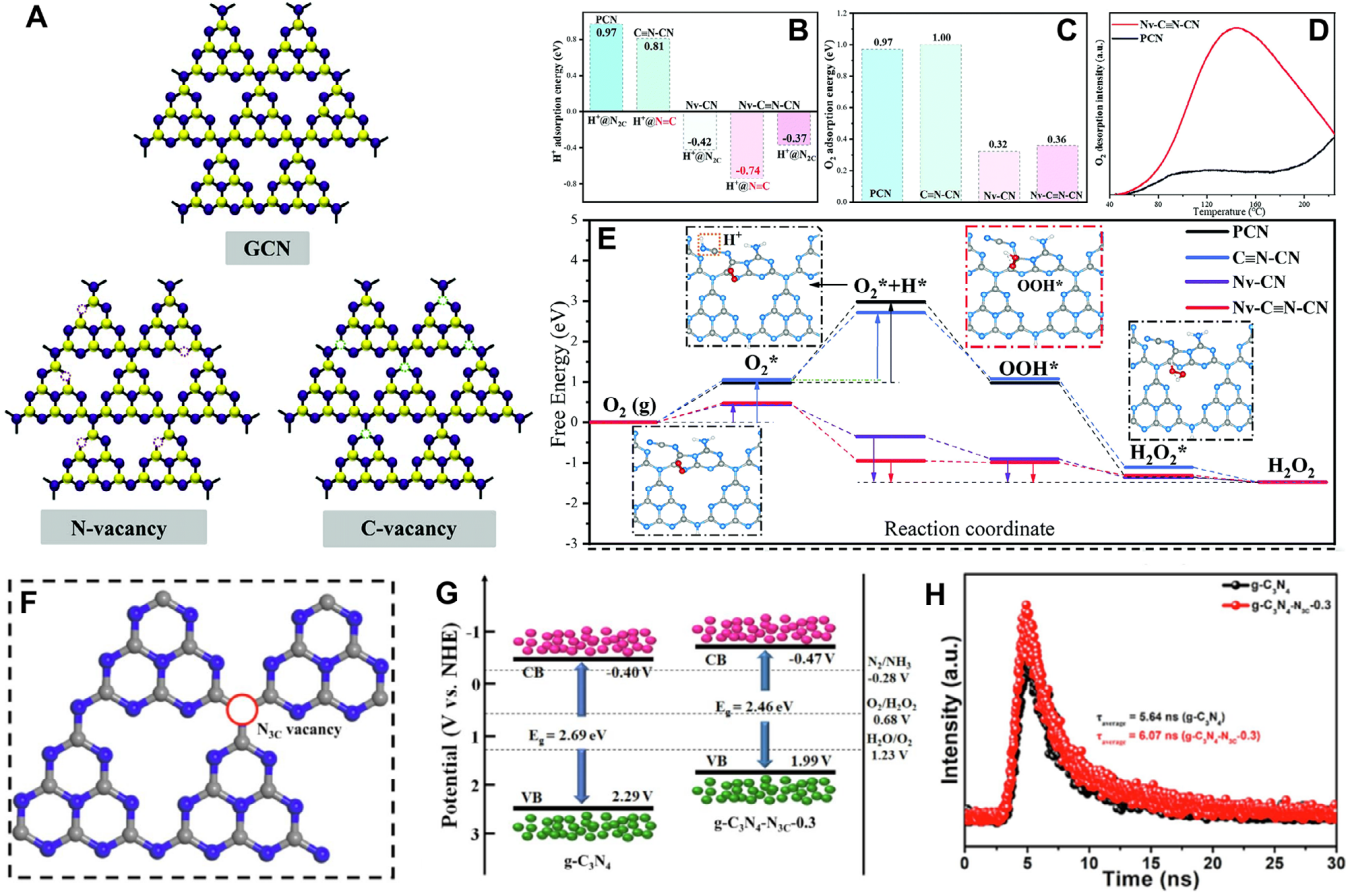
(A) Molecular structure of g‐C_3_N_4_ (GCN) with or without C_V_ and N_V_. Reproduced with permission [114]. Copyright 2021, Royal Society of Chemistry. The adsorption energy of (B) H^+^ and (C) O_2_ on g‐C_3_N_4_ with different defects. (D) Temperature‐programmed O_2_ desorption (TPD‐O_2_) profiles of g‐C_3_N_4_ with different defects. (E) Free energy profiles for O_2_ reduction to form H_2_O_2_ over different g‐C_3_N_4_ catalysts. Reproduced with permission [74]. Copyright 2022, Royal Society of Chemistry. (F) Structural model of g‐C_3_N_4_ with N_3C_ defects. (G) Diagrams of band structure. (H) Time‐resolved photoluminescence spectra of g‐C_3_N_4_ with/without N_3C_ defects. Reproduced with permission [115]. Copyright 2023, Elsevier.

Specifically for g‐C_3_N_4_ composed of a C–N heterocyclic structure, the common defects mainly include C and N_V_, which are formed by subtracting partial C or N atoms in g‐C_3_N_4_ frameworks (Figure 4A) [112]. A variety of studies have demonstrated the critical roles of C/N_V_ on g‐C_3_N_4_ for boosting H_2_O_2_ synthesis via ORR. Thermal annealing in oxygen‐deficient environments is a critical method for creating vacancies in g‐C_3_N_4_ [32, 112]. For example, the preparation of C vacancies (C_V_)–modified g‐C_3_N_4_ usually relies on heating pristine g‐C_3_N_4_ at 500–650 °C in an Ar or NH_3_ gas atmosphere. Co‐thermal treatment of the precursors of g‐C_3_N_4_ (e.g., melamine) with oxidizing bases or organics is a more facile approach. The presence of C/N_V_ is usually characterized by the EPR technique; for instance, the signal of C_V_ owns a *g* factor of 2.004 [113].

#### 3.2.1 Nitrogen Vacancies

The presence of N_V_ would affect the electronic structure of g‐C_3_N_4_. Especially, it could introduce defect energy levels close to the CB of g‐C_3_N_4_. Upon excitation of g‐C_3_N_4_, the N_V_ can accept photogenerated e^−^ in the excited state of CB, thus suppressing the rapid charge recombination. With excess e^−^, the N_V_ can serve as excellent sites for ORR by providing strong adsorption affinity to reactants such as O_2_, as well as abundant e^−^ to induce the following reduction reactions. The N atoms in g‐C_3_N_4_ mainly include three binding environments – sp^2^‐hybridized aromatic N (C═N–C), tertiary nitrogen (N‐(C)_3_) groups, and amino functional groups(C–N–H). Among them, loss of N atoms in the C═N–C environment is very popular for the creation of N_V_ (denoted as N_V_‐_2C_), and recently N_V_ (denoted as N_V_‐_3C_) derived from loss of N in N‐(C)_3_ has been reported too.

Zhang et al. introduce N_V_ into g‐C_3_N_4_ with rich cyano groups (Nv‐C≡N‐CN) by calcination in a tube furnace under Ar atmosphere [74]. Theoretical calculations reveal that the N_V_ is a favorable site for the adsorption of both reactants of H^+^ and O_2_ for H_2_O_2_ formation (Figure 4B,C). The corresponding O_2_ adsorption energy on N_V_‐modified g‐C_3_N_4_ (0.32 eV) is much lower than that on pristine g‐C_3_N_4_ and (0.97 eV) and cyano‐rich g‐C_3_N_4_ (1.00 eV), indicating that the existence of Nv can efficiently adsorb O_2_. The O_2_ adsorption capacity of g‐C_3_N_4_ was obviously enhanced with the presence of N_V_ (Figure 4D). Once adsorbed on the N_V_, the bond length of O_2_ exhibits a remarkable increase, suggesting the activation of O_2_ by the N_V_. The O_2_ reduction is thermodynamically more favorable on Nv‐C≡N‐CN to form OOH* intermediate and finally H_2_O_2_ compared with pristine g‐C_3_N_4_ (Figure 4E). This Nv‐C≡N‐CN photocatalyst shows a H_2_O_2_ generation rate of 137 µmol g^−1^ h^−1^ and achieved an AQY of 1.8% at 420 nm in pure water.

In another report, Li et al. prepared g‐C_3_N_4_ with rich N_V_‐_2C_ and cyano groups (denoted as Nv‐M) through calcination of urea accompanied with KOH [52]. As demonstrated by theoretical calculations, the melon units of pristine g‐C_3_N_4_ display an equal distribution of density of charge states due to the highly symmetric planar structure. With the presence of N_V_ and cyano groups, redistribution of charge density occurs for Nv‐M to induce electron‐rich regions, and the charge accumulation on CB brings down its potential. Importantly, the localization of charge density distributions caused by N_V_ contributes to the formation of high‐ and low‐density regions of valence e^−^, namely, the formation of an internal electric field (IEF). Hence, the rapid recombination of charge carriers can be effectively inhibited. In addition, the N_V_ exhibits a strong adsorption ability for O_2_ with an adsorption energy of −0.22 eV, which is more negative compared to that of cyano group sites (−0.15 eV) and pristine g‐C_3_N_4_ (0.76 eV). The production rate of H_2_O_2_ for Nv‐M reaches is about 7 fold higher than that of pristine g‐C_3_N_4_.

Xue et al. prepared g‐C_3_N_4_ with three‐coordinate nitrogen (N_3C_) vacancies (N_V_‐_3C_) by a simple in situ copyrolysis method (Figure 4F) [115]. Due to the introduction of N_V_‐_3C_, the semiconductive properties of g‐C_3_N_4_ exhibit a series of advancements: (1) the band gap of g‐C_3_N_4_ was narrowed from 2.69 to 2.46 eV, indicating an enhanced visible‐light‐harvesting efficiency (Figure 4G); (2) the lifetime of charge carriers was enlarged from 5.64 to 6.07 ns, suggesting the suppressed charge recombination process (Figure 4H); and (3) the charge separation and transfer rate was also accelerated. These optimizations brought by N_V_ allow the g‐C_3_N_4_ to supply abundant e^−^ for ORR and thereby efficient H_2_O_2_ synthesis.

#### 3.2.2 Carbon Vacancies

With similar effects as N_V_, the occurrence of C_V_ can also influence the electronic structures of g‐C_3_N_4_. The C_V_ breaks the C–N bond and reduces the structural symmetry of g‐C_3_N_4_ to induce unsaturated N atoms. Accordingly, the VB energy of g‐C_3_N_4_ is increased by C_V_, leading to excitation of more e^−^ and a narrowed bandgap. The unsaturated N atoms could be paramagnetic centers to attract the CB electrons, inducing the formation of electron delocalization for realizing efficient charge separation. That is, photogenerated electrons tend to accumulate in the C_V_. In addition, the C_V_ also shows superior adsorption affinity for small gas molecules such as O_2_, which could favor the subsequent reduction reactions.

**FIGURE 5:**
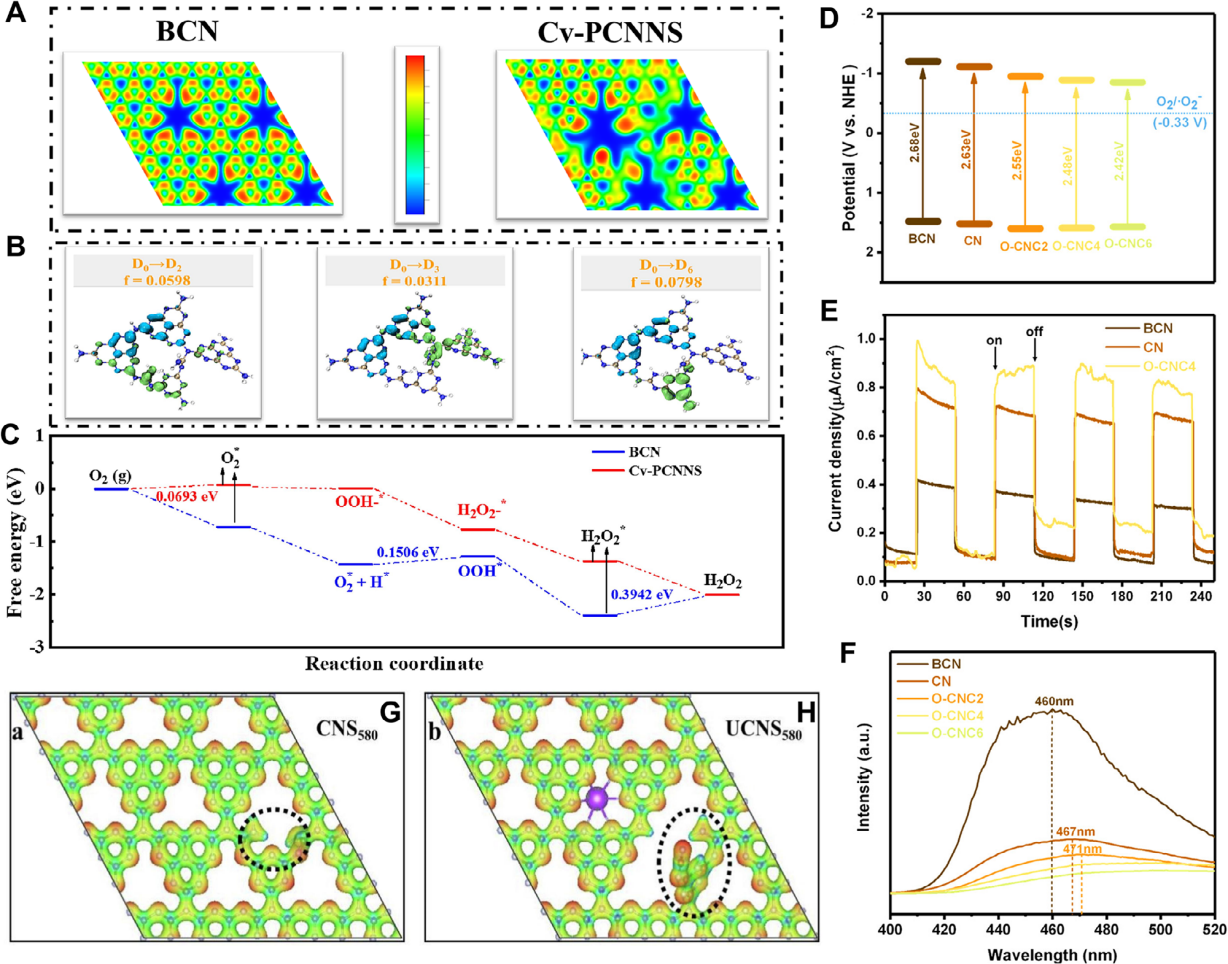
(A) Electron density maps for bulk g‐C_3_N_4_ (BCN) and porous g‐C_3_N_4_ with C_V_ (Cv‐PCNNS). (B) Electrons (green) and holes (blue) distributions for Cv‐PCNNS in terms of D_0_ → D_2_, D_0_ → D_3_, and D_0_ → D_6_ excitations, and the *f* values represent the corresponding oscillator strength. (C) ORR free‐energy diagrams for H_2_O_2_ generation over BCN and Cv‐PCNNS. Reproduced with permission [50]. Copyright 2023, Elsevier. (D) Band structure diagrams. (E) Transient photocurrent responses and (F) PL spectra of bulk g‐C_3_N_4_ (BCN) and O‐doped g‐C_3_N_4_ with C_V_ (O‐CNC*_x_*). Reproduced with permission [49]. Copyright 2021, American Chemical Society. (G) and (H) Electrostatic potential (ESP) surface distribution of g‐C_3_N_4_ with C_V_ (*G*) and g‐C_3_N_4_ with both C_V_ and cyano group (H). Reproduced with permission [51]. Copyright 2023, Elsevier.

There have been a series of recent studies focusing on H_2_O_2_ photosynthesis over C_V_‐modified g‐C_3_N_4_ photocatalysts. Li et al. synthesized porous g‐C_3_N_4_ nanosheets with C_V_ (Cv‐PCNNS) by calcination of bulk g‐C_3_N_4_ in an Ar atmosphere [50]. Density functional theory (DFT) calculations show the uniform delocalization of charge on heptazine units of pristine g‐C_3_N_4_, while charge redistribution can be observed on Cv‐PCNNS (Figure 5A), induced by the asymmetrical structure. The C_V_ induces a more localized electron density distribution and therefore improved charge separation efficiency for Cv‐PCNNS (Figure 5B). The C_V_ in Cv‐PCNNS can also favor the ORR thermodynamics (Figure 5C). The photocatalytic performance of Cv‐PCNNS reaches 984.8 µmol L^−1^ h^−1^, 10‐fold higher than the bulk g‐C_3_N_4_.

The C_V_ can also work with other modification methods to achieve synergistic promotion of the photocatalytic activity of g‐C_3_N_4_. For example, Xie et al. prepared modified g‐C_3_N_4_ (O‐CNC) with both C_V_ and oxygen doping through a microwave heating method [49]. The O‐doping, creation of C_V_, as well as mesoporous structure synergistically enhance the H_2_O_2_ production by tuning the band structure (Figure 5D), elevating charge separation efficiency (Figure 5E,F), and increasing the surface areas. Thus, the H_2_O_2_ photosynthesis rate for O‐CNC reaches 2008.4 µmol h^−1^ g^−1^, more than 4 fold higher than that of pristine g‐C_3_N_4_. Shi et al. prepared ultrathin g‐C_3_N_4_ with both C_V_ and cyano groups through a KOH‐assisted two‐step calcination method [51]. Simultaneously attaching C_V_ and cyano groups onto g‐C_3_N_4_ could strengthen the light absorption capacity. More importantly, the presence of the cyano group near the C_V_ can decrease the electron density around the C_V_, and the cyano group itself becomes the new electron‐rich center (Figure 5G,H). Hence, the photoinduced e^−^ shall tend to migrate from C_V_ to cyano groups, thus optimizing the charge separation and migration ability. This synergistic effect cannot be realized by simplex C_V_ or cyano groups. Thereby, a 42.6‐fold enhancement has been achieved for the multi‐optimized photocatalyst in terms of H_2_O_2_ generation compared with pristine g‐C_3_N_4_.

Regarding the multi‐functions of N_V_ and C_V_ for improving photocatalytic H_2_O_2_ synthesis over g‐C_3_N_4_, the similarities of N_V_ and C_V_ mainly include (1) enhancing charge separation by introducing defect states to trap e^−^ and reduce charge recombination; (2) optimizing the band structure of g‐C_3_N_4_ to facilitate more favorable redox potentials for H_2_O_2_ generation; and (3) providing key active sites for O_2_ adsorption and reduction. The N_V_/C_V_ also shows significant differences in their electronic effects and reaction mechanisms for H_2_O_2_ generation. The C_V_ in g‐C_3_N_4_ creates electron‐deficient sites, while the N_V_ generates electron‐rich sites. Based on the distinct electronic effects, C_V_ are more effective in tuning the band gap compared with N_V_, and 2e^−^ ORR is more likely to occur in the presence of C_V_, thereby boosting the ORR selectivity [72, 116, 117].

### 3.3 Junction Construction

Fast charge recombination in the bulk or at the surface of single‐phase photocatalysts like g‐C_3_N_4_ remains a critical limitation for photocatalytic activity. The construction of g‐C_3_N_4_‐based heterostructures addresses this challenge by enabling efficient charge transfer between components, which spatially separates redox centers and significantly enhances charge separation efficiency [111, 118]. Beyond improving charge dynamics, these heterostructures offer multiple synergistic advantages: (1) bandgap modulation through coupling with narrow‐bandgap semiconductors (e.g., CdS, BiVO_4_) extends light absorption into visible/NIR regions; (2) interface‐induced defect states create additional transition levels for sub‐bandgap photon utilization; and (3) surface heterojunctions with carbon materials enhance light harvesting through trapping effects while further promoting charge separation. Additional benefits include increased active sites, expanded surface area, and reduced reaction overpotentials. Based on their charge transfer mechanisms and compositions, g‐C_3_N_4_‐based heterostructures can be classified into several types, including Type II, Z‐scheme, and S‐scheme heterojunctions, as well as metal‐modified and homojunction variants. This comprehensive junction engineering approach has emerged as a powerful strategy for developing highly efficient g‐C_3_N_4_‐based photocatalysts.

#### 3.3.1 Type II Heterojunction

**FIGURE 6:**
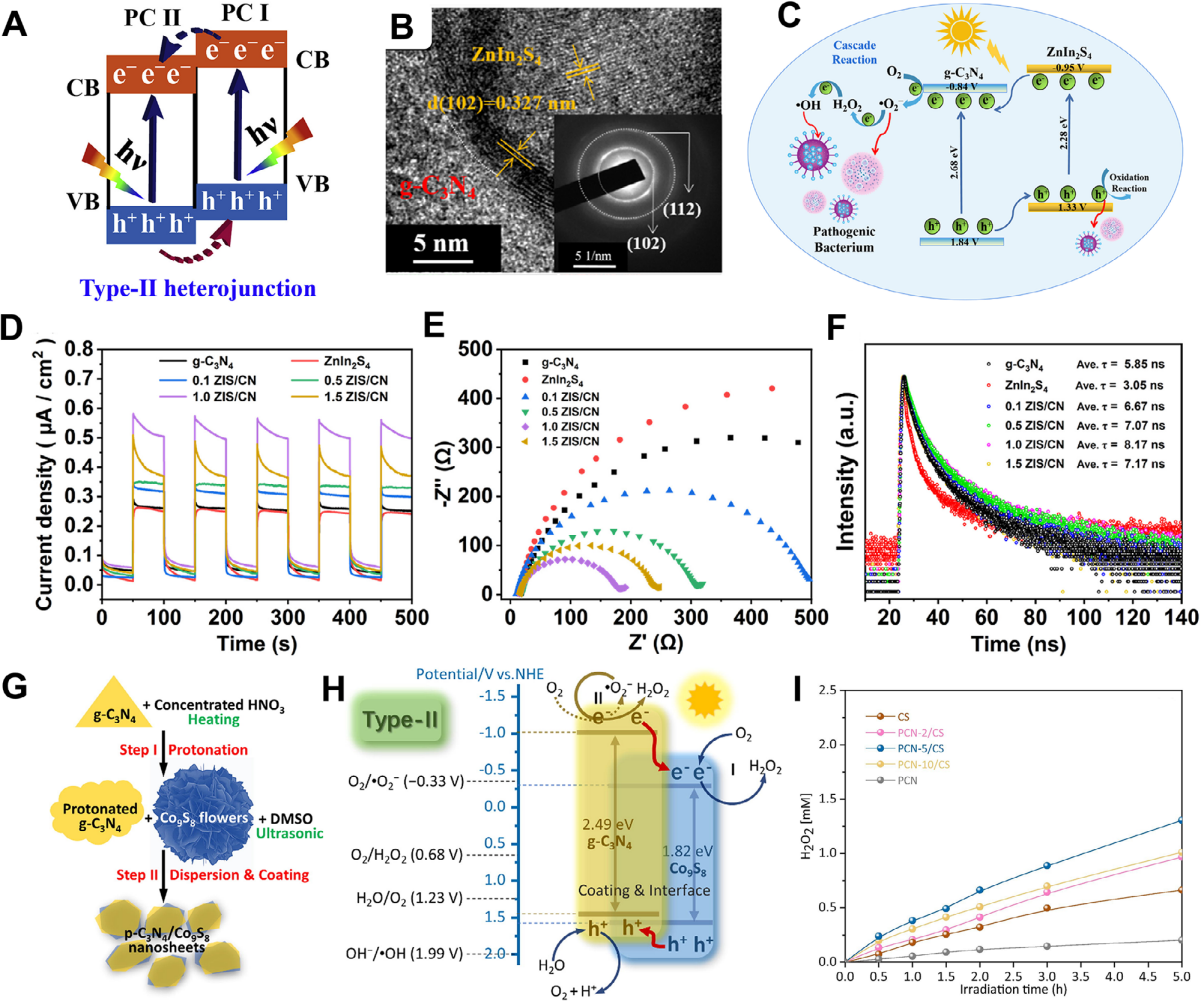
(A) The photocatalytic mechanism within a traditional type‐II heterojunction. Reproduced with permission [122]. Copyright 2020, Elsevier. (B) High‐resolution transmission electron microscopy (HRTEM) image with inset selected area electron diffraction (SAED) image of ZIS/CN composite. (C) The transfer route of photo‐induced charge carriers within ZIS/CN for H_2_O_2_ generation. (D) Transient photocurrent responses. (E) Electrochemical impedance spectroscopy (EIS) spectra. (F) Time‐resolved PL spectra of ZIS, CN, and ZIS/CN composite. Reproduced with permission [57]. Copyright 2022, Elsevier. (G) Demonstration for the synthesis of protonated g‐C_3_N_4_ coated with Co_9_S_8_. (H) Schematic illustration of the type‐II charge transfer mechanism for g‐C_3_N_4_/Co_9_S_8_ heterojunction. (I) The photocatalytic performance of g‐C_3_N_4_, Co_9_S_8_, and different g‐C_3_N_4_/Co_9_S_8_ composites on H_2_O_2_ generation. Reproduced with permission [84]. Copyright 2022, Elsevier.

For pure g‐C_3_N_4_ photocatalysts, the photogenerated charge carriers tend to recombine, thereby strongly limiting the kinetics of photocatalytic reactions. Through the construction of a unique contact interface between g‐C_3_N_4_ and other photocatalysts with staggered energy band structures, spatial separation of charge carriers can be realized by efficient charge transfer across the interface between the two photocatalysts [119, 120]. Figure 6A shows a traditional type‐II mechanism: photogenerated e^−^ and h^+^ reversely transfer from one photocatalyst to another, following the trend from larger potential to lower potential. Accordingly, the e^−^ and h^+^ are separated to different photocatalysts, leading to reduced recombination opportunities and increased lifetimes of them. In terms of the selection of semiconductors coupled with g‐C_3_N_4_, the suitability of the band structure is usually the primary consideration. The g‐C_3_N_4_ generally owns a sufficiently negative CB potential (−1.3 V vs. NHE), but a moderate VB potential (+1.4 V vs. NHE) [121]. Therefore, it's easy to construct a type II g‐C_3_N_4_‐based heterojunction with g‐C_3_N_4_ acting as photocatalyst I (PC I) in Figure 6A.

Shao et al. designed a 2D/2D ZnIn_2_S_4_/g‐C_3_N_4_ heterojunction (ZIS/CN) through an in situ growth method (Figure 6B) [57]. Vertical decoration of g‐C_3_N_4_ nanosheets with petal‐like ZnIn_2_S_4_ nanoflakes builds numerous hetero‐interfaces with potential gradients within the two components. Hence, the photogenerated charge carriers could efficiently transfer in a type II mechanism due to the relative band structure difference between ZnIn_2_S_4_ and g‐C_3_N_4_ (Figure 6C). This has contributed to enhanced charge separation and transfer efficiency for ZIS/CN (Figure 6D,E), and the lifetime of photoinduced charge carriers has increased to 8.17 ns, much larger than that of bare ZnIn_2_S_4_ (3.05 ns) and g‐C_3_N_4_ (5.85 ns) (Figure 6F). Zhang et al. constructed a type‐II heterojunction by using protonated g‐C_3_N_4_ coated with Co_9_S_8_ semiconductor for H_2_O_2_ synthesis (Figure 6G) [84]. They uniformly spread ultrathin g‐C_3_N_4_ on the surface of dispersed Co_9_S_8_ nanosheets. Besides the promotion of electron transportability and charge separation, the type‐II heterojunction opens another channel for H_2_O_2_ generation – the electrons accumulated in the CB of Co_9_S_8_ result in the dominant channel for H_2_O_2_ production via a two‐electron reduction pathway (Figure 6H). The g‐C_3_N_4_/Co_9_S_8_ composite photocatalysts deliver a H_2_O_2_ yield (2.17 mM in 5 h) much higher than that of either g‐C_3_N_4_ or Co_9_S_8_ (Figure 6I).

#### 3.3.2 Z‐Scheme Heterojunction

In photocatalysis, the redox capacities of photogenerated holes and electrons are determined by the potentials of CB and VB of semiconductors. The more negative CB and positive VB represent stronger reduction and oxidation abilities of e^−^ and h^+^, respectively. Photocatalysts with both much negative CB and positive VB could exhibit a higher photocatalytic activity. Nevertheless, the corresponding bandgap would be very large in this case, which in turn limits the optical absorption and generation of charge carriers. Therefore, it's difficult for a single‐component photocatalyst to simultaneously exhibit strong reduction or oxidation ability. An appropriate combination of two or more semiconductors to fabricate heterojunction photocatalysts is a potential approach to address this challenge, in addition to efficient separation of photogenerated charge carriers.

**FIGURE 7:**
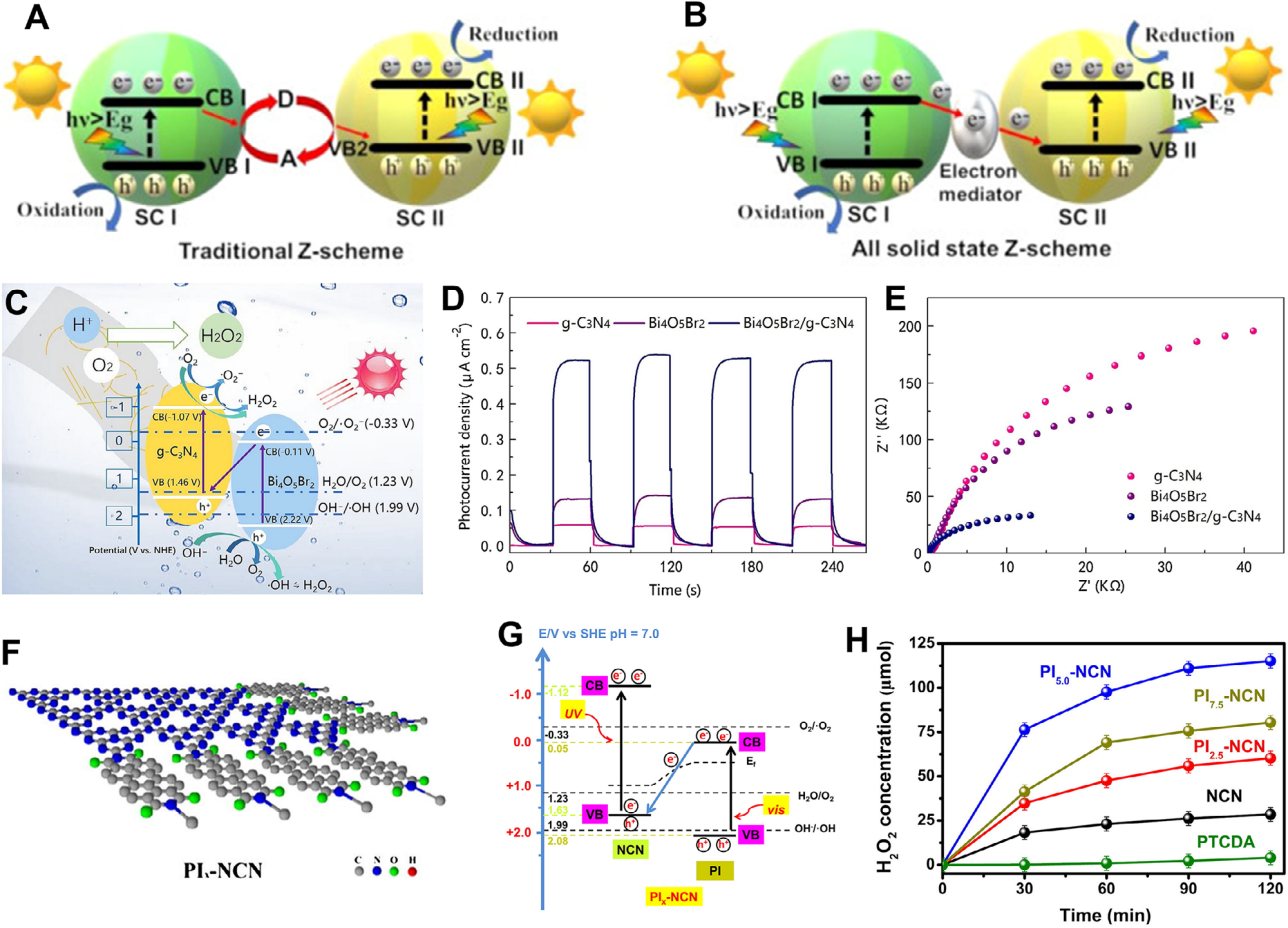
The photocatalytic mechanism for (A) traditional Z‐scheme and (B) all‐solid‐state Z‐scheme heterojunctions. Reproduced with permission [123]. Copyright 2024, Elsevier. (C) The Z‐scheme mechanism of the Bi_4_O_5_Br_2_/g‐C_3_N_4_ composite for photocatalytic H_2_O_2_ generation. (D) Transient photocurrent responses, (E) EIS spectra of g‐C_3_N_4_, Bi_4_O_5_Br_2_, and Bi_4_O_5_Br_2_/g‐C_3_N_4_. Reproduced with permission [37]. Copyright 2020, Elsevier. (F) Schematic illustration of the structure for perylene imides (PI) on g‐C_3_N_4_ nanosheets (NCN) (PI‐NCN). (G) Band structures of PI and NCN and the Z‐scheme charge transfer mechanism. (H) Comparison of the photocatalytic H_2_O_2_ generation activity. Reproduced with permission [77]. Copyright 2017, Elsevier.

The traditional type II heterojunction, though effectively enhancing the charge separation, results in a decrease of reduction and oxidation abilities of heterojunction systems from the thermodynamic perspective because of the accumulation of e^−^ and h^+^ in less negative and positive CB and VB, respectively. To address this issue, a Z‐scheme heterojunction has been developed. Figure 7A,B shows the charge transfer mode between two semiconductors in a Z‐scheme heterojunction, with or without electron mediators. The photogenerated e^−^ first transferred from the less negative CB of PC II to the electron mediator, followed by the VB of PC I for recombination with the h^+^ therein. By this, the charge carriers of PC I and PC II are effectively separated, while the e^−^ and h^+^ with high redox capacities are retained in PC I and PC II, respectively. Moreover, the electron mediator could be unnecessary for two closely contacted photocatalysts, which allows the direct charge transfer between two photocatalysts.

Extensive g‐C_3_N_4_‐based Z‐scheme heterojunctions have been studied for boosting photocatalytic H_2_O_2_ synthesis. A series of popular photocatalysts can be coupled with g‐C_3_N_4_, such as Fe_2_O_3_ [81], W_18_O_49_ [54], Bi_4_O_5_Br_2_ [37], zinc polyphthalocyanine (ZnPPc) [76], etc. The coupling of photocatalyst with strongly oxidative h^+^ is critical for elevating the oxidation ability of g‐C_3_N_4_‐based systems. Zhao et al. prepared a Bi_4_O_5_Br_2_/g‐C_3_N_4_ heterojunction by a simple water‐induced self‐assembly method to achieve a face‐to‐face connection between Bi_4_O_5_Br_2_ nanorods and g‐C_3_N_4_ nanosheets [37]. The VB potential of Bi_4_O_5_Br_2_ (2.22 V vs. NHE) is much higher than that of g‐C_3_N_4_ (1.46 V vs. NHE), while the CB potential of g‐C_3_N_4_ (−1.07 V vs. NHE) is much more negative than that of Bi_4_O_5_Br_2_ (−0.11 V vs. NHE). Through the formation of a Z‐scheme heterostructure, the composite catalyst maintains a powerful redox capacity, an oxidation potential of 2.22 V, together with a reduction potential of −1.07 V (Figure 7C). Therefore, both the water oxidation and ORR become quite favorable in terms of thermodynamics, while the enhanced charge separation efficiency and charge transfer rate promote reaction kinetics (Figure 7D,E). Accordingly, the Bi_4_O_5_Br_2_/g‐C_3_N_4_ heterojunction achieves an activity almost 25 times higher than that of g‐C_3_N_4_ in terms of H_2_O_2_ synthesis.

To construct a tight connection between two components in the heterojunction, organic semiconductors can be directly bonded to the terminal ‐NH_2_ group of g‐C_3_N_4_. For instance, Yang et al. assembled perylene imide (PI) to the edge of ultrathin g‐C_3_N_4_ nanosheets (NCN) through the reaction between perylene tetracarboxylic dianhydride and the terminal –NH_2_ from g‐C_3_N_4_ (Figure 7F) [77]. Due to the staggered band structure between PI and NCN, a Z‐scheme charge transfer route is formed to spatially isolate the oxidation and reduction reaction sites (Figure 7G), thus leading to increased charge separation efficiency. Importantly, the assembly of PI induces another pathway of water oxidation for producing H_2_O_2_, instead of the single ORR route. This is because the highly oxidative h^+^ of PI remains in the VB, which is thermodynamically favorable to trigger the water oxidation and generate •OH and subsequently H_2_O_2_. By this, the PI enhances the H_2_O_2_ yield over NCN by up to four times (Figure 7H).

#### 3.3.3 S‐Scheme Heterojunction

**FIGURE 8:**
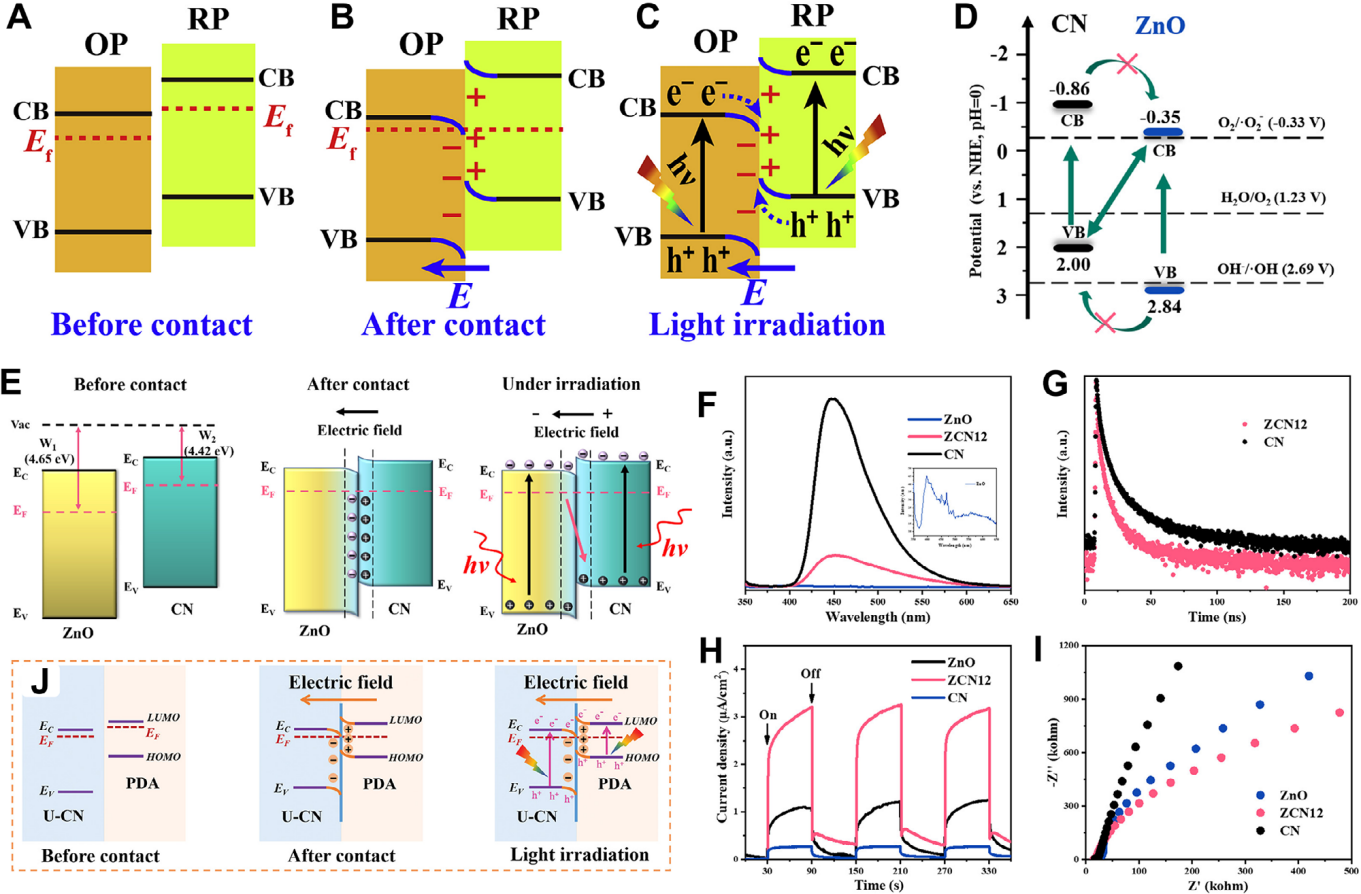
(A–C) The S‐scheme mechanism for heterostructure photocatalysts. Reproduced with permission [122]. Copyright 2020, Elsevier. (D) Band structure diagrams for g‐C_3_N_4_ (CN) and ZnO. (E) The formation of IEF and band edge bending at the interface of CN and ZnO in contact and the illustration of the S‐scheme charge transfer route under light irradiation. (F) Steady‐state PL spectra, (G) time‐resolved PL spectra, (H) transient photocurrent responses, and (I) EIS spectra of ZnO, CN, and ZnO/CN composite. Reproduced with permission [61]. Copyright 2021, American Chemical Society. (J) Schematic illustration of the S‐scheme photocatalytic mechanism for the heterostructure of PDA and U‐CN. Reproduced with permission [58]. Copyright 2023, Wiley.

Although the Z‐scheme mechanism has been widely studied, it still faces limitations in achieving efficient charge transfer. In an ideal situation, the e^−^ transfer from the CB of PC II to the VB of PC I induces recombination, leaving the e^−^ of PC I and h^+^ of PC II for surface redox reactions. Nevertheless, the e^−^–h^+^ pairs with higher redox capacities are more likely to quench each other because of larger thermodynamic driving forces. Hence, a more recent type of S‐scheme photocatalytic mechanism was proposed by Yu et al. in 2019 [124]. The S‐scheme heterojunction usually consists of two n‐type photocatalysts, named as reduction photocatalyst (RP) and oxidation photocatalyst (OP) (Figure 8A). The VB of OP is more positive, and the CB and Fermi level (*E*_F_) of RP are more negative. The g‐C_3_N_4_ is a good candidate for RP due to a negative‐enough CB potential, and various OPs can be coupled with g‐C_3_N_4_, especially the semiconductors with a high VB potential (e.g., ZnO, WO_3_, Ag_3_PO_4_, BiVO_4_) [125, 126]. Once the RP and OP contact each other, the difference in *E*_F_ will drive the e^−^ transfer from the CB of the RP to that of the OP until the matching of two *E*_F_ (Figure 8B) [122]. Such an e^−^ transfer pathway could induce an IEF and band bending at the contact interface, thus contributing to efficient charge carrier transfer (Figure 8C).

To date, various photocatalysts have been used for preparing g‐C_3_N_4_‐based S‐scheme heterojunction to enhance the photocatalytic activity for H_2_O_2_ generation [11]. Metal oxides such as ZnO and TiO_2_ with high VB potentials are good candidates in this context (Figure 8D). Liu et al. designed hierarchically porous ZnO/g‐C_3_N_4_ S‐scheme heterojunction photocatalysts by using a two‐step calcination method with ZIF‐8 and urea as precursors [61]. Figure 8E shows the S‐scheme charge transfer mechanism in the ZnO/g‐C_3_N_4_ heterojunction. As the work function of ZnO (4.65 eV) is higher than that of g‐C_3_N_4_ (CN, 4.42 eV), the e^−^ will spontaneously transfer from CN to ZnO until their *E*_F_ reaches equilibrium when the two semiconductors are in close contact in the dark. As a result, the CN and ZnO become positively and negatively charged, inducing the formation of an IEF at the contact interface. The band edge of CN bends upward, while the band edge of ZnO bends downward. When subjected to light radiation, the IEF and bending of the band edge work together to drive the recombination of the e^−^ in the CB of ZnO with the h^+^ in VB of CN, thus preserving the e^−^ in the CB of CN and h^+^ in the VB of ZnO for reduction and oxidation reactions. The PL characterization confirms the inhibited charge recombination process and a higher lifetime of charge carriers (Figure 8F,G), while the photocurrent responses and EIS spectra show the enhanced charge separation and transfer (Figure 8H,I). Given a high charge separation efficiency and reservation of the strong redox capacities, the H_2_O_2_ generation rate for ZnO/g‐C_3_N_4_ is enhanced by 3.4 and 5.0 times compared with pure CN and ZnO, respectively.

Organic semiconductors such as polymers and metal‐organic frameworks can also be coupled with g‐C_3_N_4_ to form an S‐scheme heterojunction. The organic semiconductors with a more negative CB potential could further enhance the reduction capacity in a g‐C_3_N_4_‐based S‐scheme heterojunction. Zhang et al. coupled polydopamine (PDA) with ultrathin g‐C_3_N_4_ (U‐CN) to construct an S‐scheme heterostructure through in situ self‐polymerization [58]. Given a lower work function of PDA (4.36 eV) compared with U‐CN (4.51 eV), the free e^−^ would spontaneously transfer from PDA to U‐CN to reach equal *E*_F_ once they are in close contact (Figure 8J). Accordingly, the IEF is formed, directing from PDA to U‐CN. When excited by irradiation, the IEF would induce the recombination of e^−^ in CB of U‐CN with h^+^ in VB of PDA. Hence, the photogenerated charge carriers are effectively separated, and the strongly reductive e^−^ from PDA is available for ORR to produce H_2_O_2_. The optimal U‐CN/PDA photocatalyst could generate H_2_O_2_ at a rate of 3801.25 µmol g^−1^ h^−1^ under light irradiation, which is around 11 and 2 times that of single U‐CN and PDA, respectively. Similarly, Xia et al. grafted supramolecular zinc porphyrin (Zn‐TCPP) on g‐C_3_N_4_ (CN) into an S‐scheme heterojunction via calcination to form a –CONH– bridging bond [60]. The H_2_O_2_ synthesis rate by Zn‐TCPP/CN composites (591.9 µmol g^−1^ h^−1^) is 3.1 and 9.0 times higher compared with CN and Zn‐TCPP, respectively. As the work function of CN was larger compared with Zn‐TCPP, the IEF shows a direction from Zn‐TCPP to CN. Thus, the photogenerated e^−^ of CN recombines with h^+^ of Zn‐TCPP, and the ORR reduction sites locate on CB of Zn‐TCPP.

#### 3.3.4 Homojunction

Despite the strong interests in the development of g‐C_3_N_4_‐based heterojunctions, the homojunction has recently attracted increasing attention by overcoming the limitations of heterostructures on materials selection, interfacial and band structure matching, and preparation methods [127, 128]. Generally, a homojunction refers to an interface between one type or similar semiconductors with a staggered energy band structure. The difference in band structure is usually induced by different crystal phases, doping levels, microstructures, and exposed facets. The homojunctions keep the advantages of heterojunction‐based photocatalysts in efficient charge separation because of the built‐in electric field derived from different band structures. Importantly, the homojunction with similar chemical compositions can avoid the interfacial lattice mismatch by providing continuous band bonds, which is inevitable for heterojunctions coupled by different semiconductors with different lattice structure constants and atomic thermal expansion rates. Accordingly, the charge separation efficiency in homojunctions could be higher, considering the non‐negligible recombination centers of heterojunctions caused by lattice distortion and grain boundaries at the interface [129].

**FIGURE 9:**
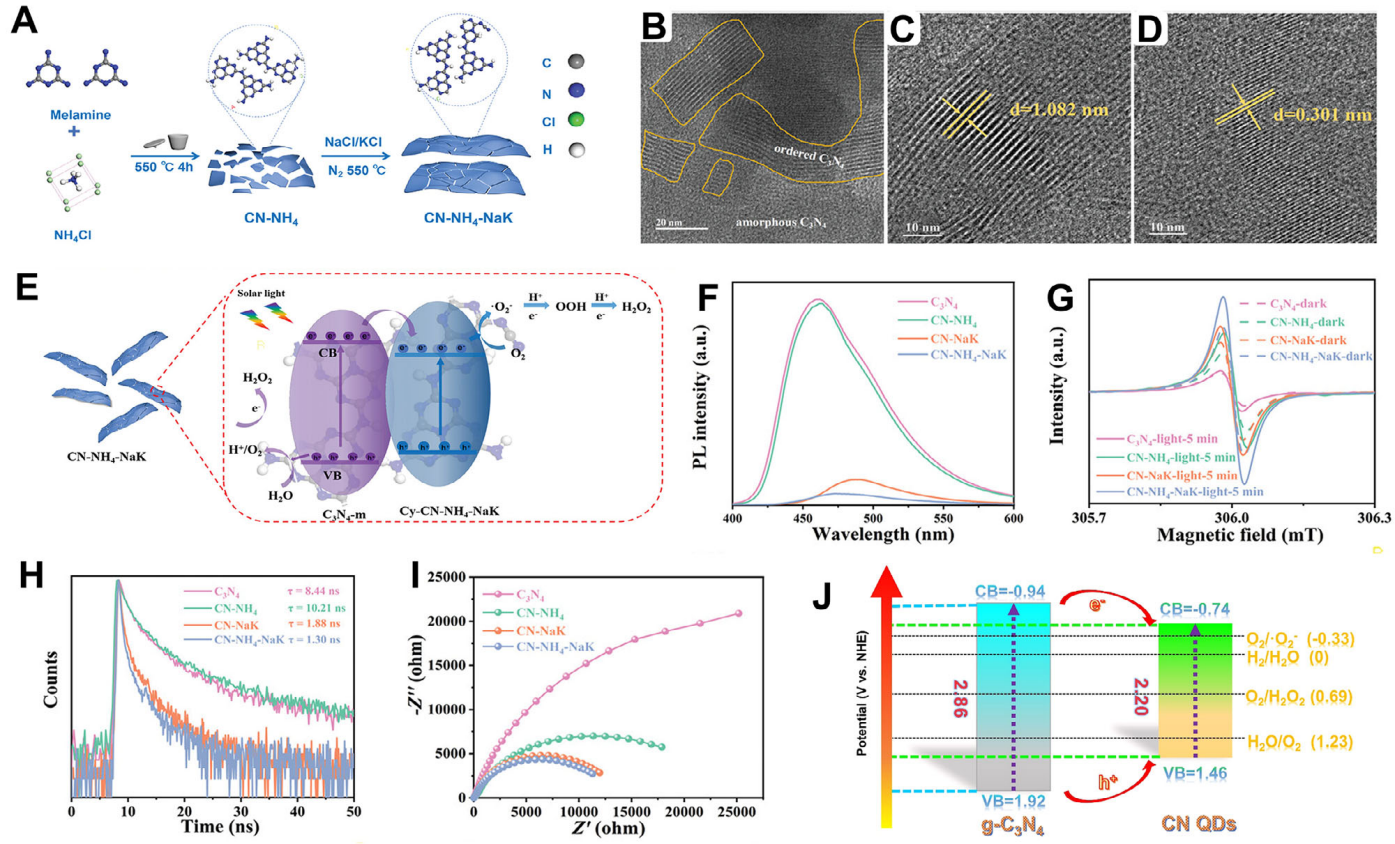
(A) The synthesis procedure of g‐C_3_N_4_‐based multi‐interfacial order–disorder homojunction (CN‐NH_4_‐NaK). (B) HRTEM and (C–D) enlarged HRTEM images of CN‐NH_4_‐NaK. (E) Schematic illustration of the mechanism for H_2_O_2_ generation on CN‐NH_4_‐NaK. (F) Steady‐state PL spectra, (G) EPR spectra of catalysts under irradiation, (H) time‐resolved PL spectra, and (I) EIS spectra of CN‐NH_4_‐NaK and other reference catalysts. Reproduced with permission [55]. Copyright 2024, Wiley. (J) The band structure of g‐C_3_N_4_ and CN QDs, and the charge transfer route in the composite photocatalyst. Reproduced with permission [83]. Copyright 2022, Elsevier.

He et al. devised an innovative approach to create a g‐C_3_N_4_ homojunction (CN‐NH_4_‐NaK) featured with multiple order–disorder interfaces through a method of fragmenting and reassembling (Figure 9A) [55]. The CN‐NH_4_‐NaK is composed mainly of well‐crystallized (Cy‐CN‐NH_4_‐NaK) and a small portion of amorphous g‐C_3_N_4_ (C_3_N_4_‐m) (Figure 9B–D). This photocatalyst delivered an excellent yield and selectivity for H_2_O_2_ photosynthesis. The H_2_O_2_ generation rate has an enhancement of 158‐fold relative to pristine g‐C_3_N_4_, which is ascribed to boosted kinetics and thermodynamics by the formation of multi‐interface homogeneous junctions. Figure 9E displays the photocatalytic mechanism of the multi‐interfacial Cy‐CN‐NH_4_‐NaK/C_3_N_4_‐m homojunction. With different crystallinity and alkaline metal doping (i.e., Na^+^ and K^+^), the two C_3_N_4_ components show obvious gaps in both CB and VB positions. The gap in CB potentials drives the transfer of photogenerated e^−^ from the amorphous C_3_N_4_‐m region to the high crystallinity and well‐ordered Cy‐CN‐NH_4_‐NaK region, thus facilitating spatial separation of redox centers and exposure of active sites. The photoelectrochemical measurements confirm the inhibited charge recombination (Figure 9F), enhanced charge separation (Figure 9G), shorter charge lifetimes (Figure 9H), and faster charge transfer (Figure 9I) for CN‐NH_4_‐NaK compared with pristine g‐C_3_N_4_. Hence, the accumulation of e^−^ in the CB of Cy‐CN‐NH_4_‐NaK facilitates ORR and H_2_O_2_ generation, while the water oxidation occurs on C_3_N_4_‐m for consuming the leaving h^+^.

Ma et al. anchored C_3_N_4_ quantum dots (CN QDs) onto 2D C_3_N_4_ nanosheets to obtain a homojunction photocatalyst [83]. Compared with pristine C_3_N_4_, the CN QDs possess a much smaller bandgap, as well as less negative CB and less positive VB potentials (Figure 9J), because of the significant size effect and quantum limiting effect. This has led to the extension of light absorption with an edge up to nearly 600 nm. Importantly, given the obvious gaps in CB and VB positions, both the photogenerated e^−^ and h^+^ of CN would transfer to CB and VB of CN QDs, respectively. Thus, enhanced charge separation efficiency can be guaranteed. The e^−^ accumulated in the CB of CN QDs could efficiently mediate ORR for H_2_O_2_ generation, while the h^+^ also concentrated in the VB of CN QDs for water oxidation (Figure 9J). This means that the CN QDs are the active sites for both reduction and oxidation reactions. The composite photocatalyst exhibits 8.6 times higher performance for H_2_O_2_ generation than that of pristine g‐C_3_N_4_, reaching 115 µmol L^−1^ h^−1^ in a pure water and visible light system.

### 3.4 Metal Cocatalyst Loading

**FIGURE 10:**
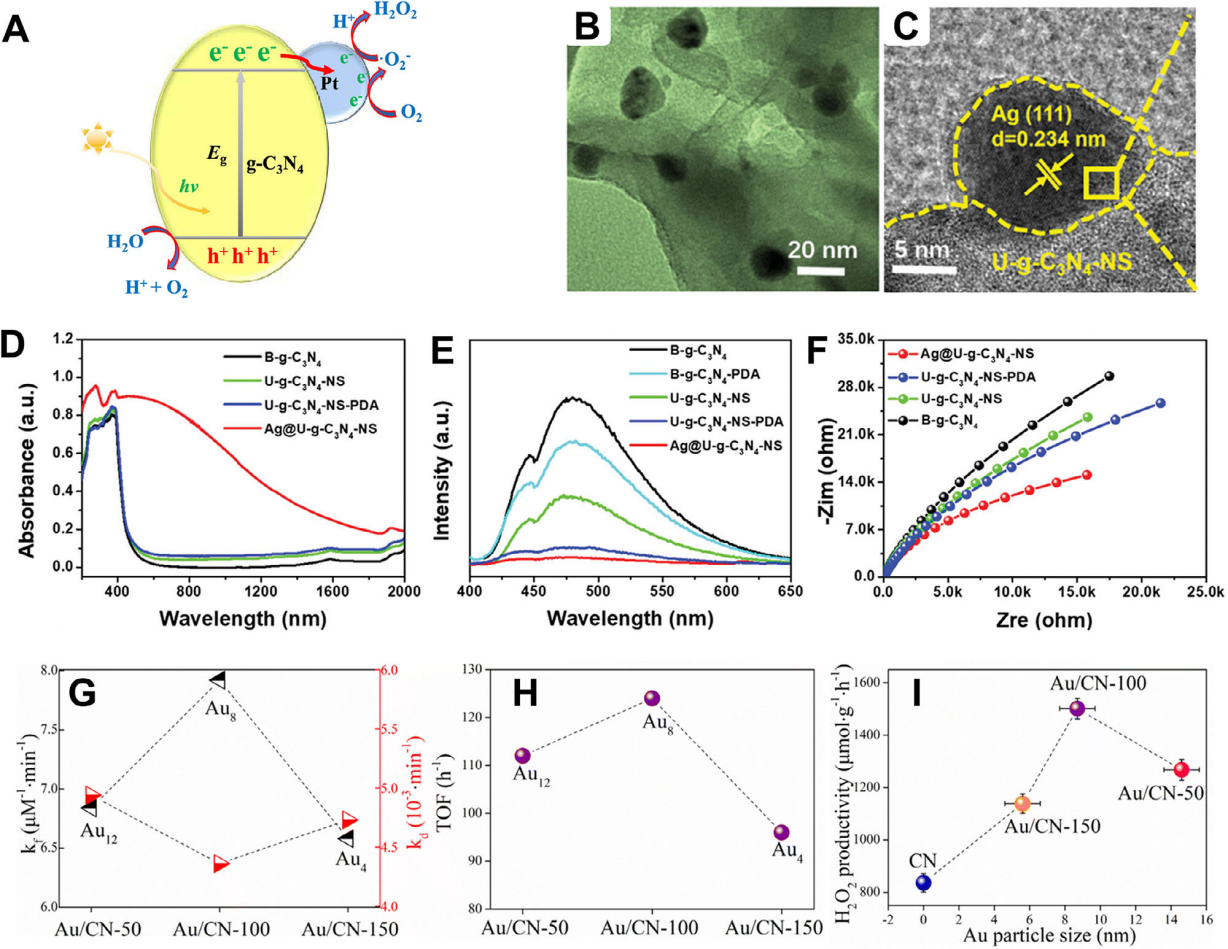
(A) The charge transfer pathway for Pt/g‐C_3_N_4_ composite photocatalyst. Reproduced with permission [136]. Copyright 2024, American Chemical Society. (B) and (C) TEM and HRTEM images of Ag@U‐g‐C_3_N_4_‐NS; (D) UV–vis DRS spectra, (E) PL spectra, and (F) EIS spectra of g‐C_3_N_4_ and Ag@U‐g‐C_3_N_4_‐NS. Reproduced with permission [137]. Copyright 2019, Wiley. (G) Comparison of the H_2_O_2_ formation (*k*_f_) and decomposition rates (*k*_d_), (H) TOF values, and (I) H_2_O_2_ productivity. Reproduced with permission [64]. Copyright 2022, Royal Society of Chemistry.

Apart from semiconductors, metal nanoparticles or clusters, especially noble metals (e.g., Ag, Au, and Pt), are also promising cocatalysts to enhance the photocatalytic performance of g­C_3_N_4_ [130, 131, 132, 133]. The different work functions between g‐C_3_N_4_ and metals could induce a contact potential difference upon their close contact, which is named as the Schottky barrier. By this, the photogenerated e^−^ would directionally migrate from the semiconductor to the metal (Figure 10A), so as to boost the charge separation efficiency [130, 134]. Furthermore, some noble (e.g., Au, Ag) and even non­noble metals (e.g., Bi) display the surface plasmon resonance (SPR) effect, which could improve the light absorption [135]. Meanwhile, the surface of noble metals can act as excellent active sites for accelerating different kinds of catalytic reactions.

Cai et al. prepared Ag‐decorated ultrathin g‐C_3_N_4_ nanosheets (Ag@U‐g‐C_3_N_4_‐NS) using a mussel‐inspired strategy [137]. The Ag nanoparticles show a discrete distribution on Ag@U‐g‐C_3_N_4_‐NS, suggesting a hierarchical heterostructure with superior dispersion (Figure 10B,C). The catalyst displays significantly enhanced H_2_O_2_ synthesis efficiency with an initial rate of 1.975 µM min^−1^ relative to the negligible activity of pristine g‐C_3_N_4_. Besides the large specific surface area, Ag nanoparticles play a key role in such an enhancement of performance. Much stronger absorption intensity (from 200–2000 nm) and mild redshift are observed for Ag@ U‐g‐C_3_N_4_‐NS relative to other catalysts, which can be related to the localized surface plasmon resonance (LSPR) of the Ag nanoparticles (Figure 10D). Moreover, the PL and EIS spectra confirm the suppressed recombination of charge carriers and facilitated transfer of electrons for photocatalytic reactions (Figure 10E,F).

The effect of different metal nanoparticle loading varies for the production of H_2_O_2_ over g‐C_3_N_4_. It has been demonstrated that loading Pt nanoparticles would inhibit the activity of g‐C_3_N_4_ for H_2_O_2_ generation due to the low selectivity of the two‐electron oxygen reduction mode induced by the electron migration pathway [138]. Moreover, the size of metal nanoparticles determines their dispersion and electronic structure, thus significantly affecting the photocatalytic activity. Song et al. dispersed a series of size‐different Au nanoparticles onto g‐C_3_N_4_, and found that there is a volcanic trend for H_2_O_2_ formation and decomposition rates and productivity when decreasing the particle size of Au (Figure 10G–I) [64]. The highest H_2_O_2_ production rate over g‐C_3_N_4_ with loading of medium‐sized Au particles (∼8.7 nm) could reach 1052 µmol g^−1^ h^−1^. The role of Au nanoparticles with an appropriate size shows various significant roles for promoting the H_2_O_2_ generation, including (1) promoting photon absorption and spatial separation of charge carriers, (2) enhancing O_2_ adsorption and providing selective two‐electron ORR sites, and (3) facilitating the desorption of H_2_O_2_ product and inhibiting H_2_O_2_ decomposition.

Besides noble metals, metal phosphides/oxides have also been widely reported as a helpful cocatalyst for promoting photocatalytic performance. Xue and coworkers reported a novel Co_x_Ni_y_P cluster incorporated P‐doped g‐C_3_N_4_ photocatalyst (Co_x_Ni_y_P‐PCN) for pure water splitting to produce H_2_O_2_ and H_2_ [139]. It was found that the chemical connection between PCN and CoNiP is reinforced due to the substitution of C in the catalyst with P. Furthermore, the surface redox potential is also optimized by cocatalyst integration. These critical effects by Co_x_Ni_y_P effectively facilitate vectorial charge transfer between PCN and CoNiP and the contingent surface mass diffusion process. Liu et al. synthesized a B‐doped g‐C_3_N_4_ (BCN) tailored with coordinatively unsaturated FeOOH and CoO*_x_* clusters (CoO*_x_*‐BCN‐FeOOH) [102]. The CoO*_x_* sites on CoO*_x_*‐BCN‐FeOOH drive hole‐mediated water oxidation and enhance electron longevity, while FeOOH accepts e^−^ and boosts oxygen activation. Moreover, the coordinatively unsaturated FeOOH can adjust the Pauling‐type adsorption configuration of O_2_, which stabilizes peroxide species and inhibits the formation of •O_2_^−^. Thus, this composite catalyst can selectively reduce O_2_ to H_2_O_2_ by the direct one‐step two‐electron reaction pathway.

### 3.5 Single Atom Loading

Single‐atom catalysts (SACs) are an emerging type of catalyst possessing notable advantages compared to traditional nanoparticle catalysts. Given the atomical metal dispersion and the unique unsaturated coordination environment, the SACs are cost‐effective and have excellent catalytic activity, selectivity, and stability toward target reactions [140, 141]. The unique C–N heterocyclic structure of g‐C_3_N_4_ makes it a good carrier of SAs. The numerous regularly arranged N atoms in the s‐triazine ring can form various coordination bonds with different metal SAs. Besides, the abundant functional groups and vacancies could ensure the rational modulation of atomic size, composition, and dispersion. Through adjustment of the species, coordination mode, and anchoring position of SAs, the electronic binding states can be regulated for improving the charge dynamics [140]. These advantages make g‐C_3_N_4_‐based SACs promising candidates in photocatalytic H_2_O_2_ synthesis.

**FIGURE 11:**
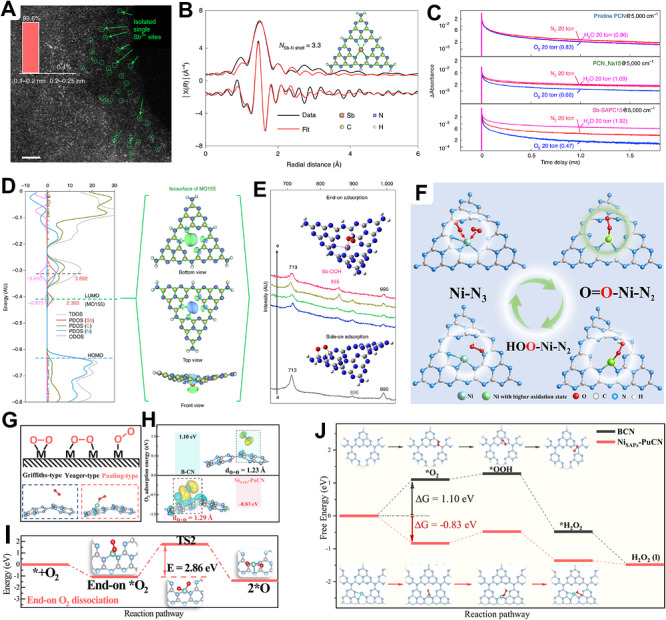
(A) High‐magnification high‐angle annular dark‐field scanning transmission electron microscopy (HAADF‐STEM) image and size distribution of Sb single atoms on g‐C_3_N_4_. (B) The EXAFS fitting result of the Sb coordination structure. (C) The comparison of transient absorption decay of catalysts under N_2_, O_2_, and H_2_O atmospheres. (D) DFT calculation of density of states (DOS) of Melem_3Sb3^+^ combined with the isosurface of LUMO. (E) Raman spectra obtained during the photoreaction of different catalysts in different solution conditions ((a–d) g‐C_3_N_4_, Sb‐SAPC1, Sb‐SAPC5, and Sb‐SAPC15 in 10% 2‐propanol aqueous solution, respectively. (e) Sb‐SAPC15 in pure water). Reproduced with permission [143]. Copyright 2021, Springer Nature. (F) Schematic illustration of H_2_O_2_ generation on the Ni sites via 2e^−^ ORR. (G) The O_2_ adsorption structure on metal sites (top) and the structure of O_2_ adsorption on BCN (bottom left) and Ni_SAPs_‐PuCN (bottom right). (H) The O_2_ adsorption energy for BCN and Ni_SAPs_‐PuCN and the corresponding charge difference density. (I) The O_2_ dissociation energies and profile for end‐on O_2_ adsorption configurations over Ni_SAPs_‐PuCN. (J) The free energy profiles of ORR for H_2_O_2_ generation over BCN and Ni_SAPs_‐PuCN. Reproduced with permission [34]. Copyright 2023, Springer Nature.

To date, a variety of single metal atoms have been loaded on g‐C_3_N_4_, such as Mn, Co, Fe, Cu, etc [142]. Recently, Sb, Ni, and Co SAs were successfully loaded on g‐C_3_N_4_ for highly efficient H_2_O_2_ synthesis [34, 92, 143]. Teng et al. used a bottom‐up method to prepare a Sb single‐atom g‐C_3_N_4_ photocatalyst (Sb‐SAPC) for the production of H_2_O_2_ [143]. The Sb SAs exhibit uniform dispersion on the surface of g‐C_3_N_4_ (Figure 11A). Extended X‐ray absorption fine structure (EXAFS) analysis reveals the coordination of each Sb atom with 3.3 N atoms on average (Figure 11B). The charge carrier dynamics were examined by time‐resolved infrared absorption spectroscopy (Figure 11C), and the results suggest that it's favorable for the reaction between O_2_ and the deeply trapped e^−^ induced by Sb sites, while the expedient transfer of h^+^ to water inhibits the charge recombination process. Moreover, the Sb contributes a new molecular orbital located at the LUMO bottom (Figure 11D), and the e^−^ aggregation on the Sb sites induces an ideal electronic configuration for O_2_ adsorption via the end‐on adsorption mode (Figure 11E). Hence, the single Sb sites could reduce O_2_ via a 2e^−^ ORR pathway to form H_2_O_2_. By this, the H_2_O_2_ generation on Sb‐SAPC reaches an AQY of 17.6% at 420 nm and a solar‐to‐chemical conversion efficiency of 0.61%.

Zhang et al. construct a high‐loading Ni single‐atom g‐C_3_N_4_ photocatalyst (NiSAPs‐PuCN) by tuning the substrate microstructure and optimizing the loading process [34]. The EXAFS fitting results show that each Ni atom in NiSAPs‐PuCN is bonded with three N atoms as Ni–N_3_ coordination with an average Ni–N bond length of about 2.07 Å (Figure 11F). Importantly, the Ni–N_3_ sites can effectively activate O_2_ molecules by evolving to form a key *OOH intermediate before finally forming HOO‐Ni‐N_2_ (Figure 11F). Theoretical calculations and experiments demonstrate 1) the thermodynamically favorable O_2_ adsorption on Ni SAs in an end‐on configuration (Figure 11G,H), 2) suppressed dissociation of the O═O bond and reduced formation energy barrier of *OOH due to the evolution of the active sites structure (Figure 11I,J). Such an innovative H_2_O_2_ evolution pathway has led to remarkably improved H_2_O_2_ production activity and selectivity, realizing an apparent quantum yield of 10.9% at 420 nm and a solar‐to‐chemical conversion efficiency of 0.82% in a pure water system.

### 3.6 Functionalization

As an organic polymeric semiconductor, g‐C_3_N_4_ can be easily modified by attaching various functional groups during the thermal polymerization process or after this. Modification of the functional group can optimize the intrinsic conjugation system, energy band structure, and photoelectrical properties of g‐C_3_N_4_. Various functional groups have been reported to be grafted into the g‐C_3_N_4_ system, such as hydroxyl, amino, cyano, and aromatic groups. Given the strong chemical bonding with g‐C_3_N_4_, the functional groups play critical roles in reducing the energy barrier of intramolecular charge transfer. Besides, these functional groups can tailor the electronic structure of g‐C_3_N_4_ and adjust its energy band structure, thus facilitating visible‐light absorption and charge separation. Amino (–NH_2_) and hydroxyl (–OH) group functionalization can modify surface electronic states, improving charge separation and light absorption. For example, NH_2_‐rich g‐C_3_N_4_ shows enhanced visible‐light response in the region of 450–800 nm due to n→π* transitions [144]. Strong electron‐withdrawing groups such as cyano (–C≡N) and carboxyl (–COOH) groups can shift the CB downward and reduce the bandgap. Moreover, π‐conjugated aromatic modifiers (e.g., phenyl, pyrene) enhance light harvesting by improving charge delocalization [145]. Simultaneously, these functional groups can also create built‐in electric fields that promote charge separation. In addition, the terminal near‐field functional groups can serve as the key active sites for adsorption and activation of reactants such as O_2_.

**FIGURE 12:**
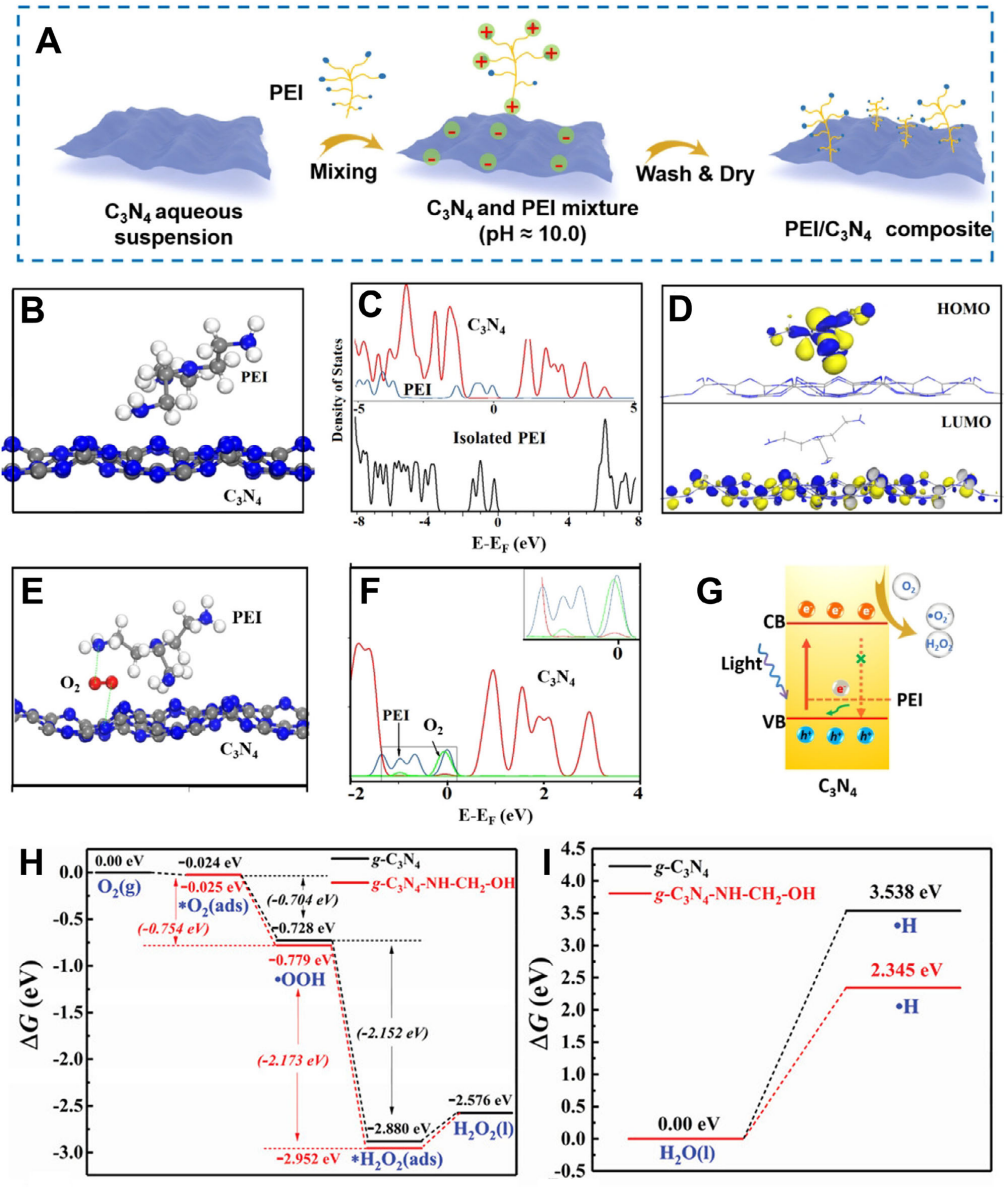
(A) Schematic illustration of the preparation of C_3_N_4_ nanosheets with PEI functionalization through the electrostatic assembly process. (B) DFT calculations for simulating the structures of single PEI adsorbed on C_3_N_4_. (C) DOS of PEI/C_3_N_4_ (top) and single PEI (down). (D) HOMO and LUMO for PEI/C_3_N_4_ composite. (E) The adsorption of O_2_ on PEI/C_3_N_4_. (F) DOS of PEI/C_3_N_4_ after the O_2_ adsorption. (G) Charge transfer route within PEI/C_3_N_4_ for ORR. Reproduced with permission [94]. Copyright 2021, American Chemical Society. Theoretical calculation of free‐energy diagrams: (H) ORR for H_2_O_2_ generation and (I) dehydrogenation of H_2_O to generate *•*H. Reproduced with permission [35]. Copyright 2022, Wiley.

Zeng et al. grafted cationic polyethylenimine (PEI) molecules onto g‐C_3_N_4_ for boosting the photocatalytic H_2_O_2_ production (Figure 12A) [94]. PEI is a cationic polyelectrolyte with rich amine groups and is thus positively charged. Hence, the PEI can be adsorbed onto the negatively charged surface of g‐C_3_N_4_ via electrostatic attraction (Figure 12A,B). It was found that PEI can tune the local electronic environment of C_3_N_4_. As presented in Figure 12C, an intermediate state was observed above the VB edge of g‐C_3_N_4_. These energy levels across the Fermi level can combine with photogenerated h^+^ in VB, thus enhancing the charge separation efficiency, as suggested by the frontier orbitals HOMO and LUMO (Figure 12D). Moreover, the O_2_ molecules can be favorably adsorbed into the middle of PEI and g‐C_3_N_4_ (Figure 12E), which induces additional interband states around the Fermi levels (Figure 12F). This is a further promotion of charge separation through the combination with photoinduced electrons (Figure 12G). Thereby, the PEI/C_3_N_4_ shows a high activity for two‐electron O_2_ reduction, and the H_2_O_2_ generation rate reaches 208.1 µmol g^−1^ h^−1^ in pure water, realizing a 25‐fold enhancement relative to pristine g‐C_3_N_4_.

Apart from the surface coating by adsorption, covalent bond binding ensures a stronger interaction between the functional groups and g‐C_3_N_4_. Gou et al. found that the introduction of cyano groups into g‐C_3_N_4_ could affect the IEF of g‐C_3_N_4_, whose value is increased from 3.38 to 4.09 [73]. Such a strengthening of IEF has led to a significant enhancement in separation efficiency for charge carriers. Further assisted by inserting Na^+^ into the interlayer, a three‐dimensional charge carrier transport channel was constructed to maximally facilitate the charge separation and transport. The optimum H_2_O_2_ formation rate constant thus reaches 160.90 µM h^−1^ for cyano‐rich g‐C_3_N_4_, 17.2 times higher than the pristine g‐C_3_N_4_. Meanwhile, the corresponding AQY was determined to be 14.52 % at 420 nm, exceeding most of the previous metal‐free photocatalysts.

In another work, Liu et al. introduced –NH‐CH_2_‐OH groups onto the surface of g‐C_3_N_4_ by making use of the selective reaction between the formaldehyde and –NH_2_ groups of g‐C_3_N_4_ [35]. It was found that the –NH‐CH_2_‐OH on the g‐C_3_N_4_ does good to the adsorption and activation of O_2_ (Figure 12H), as well as the water dehydrogenation (Figure 12I). The g‐C_3_N_4_ with –NH‐CH_2_‐OH is more powerful for capturing O_2_ molecules and locking the O_2_ with a distance (3.0852 Å) shorter than that for pristine g‐C_3_N_4_ (3.1070 Å). Furthermore, the adsorbed O_2_ displays a longer bond length of O═O in the case of O_2_/g‐C_3_N_4_ with –NH‐CH_2_‐OH configuration. Due to the advances in both thermodynamics and kinetics, this functionalized photocatalyst presents over 1280% higher activity for H_2_O_2_ production in pure water.

Besides, functionalization of g‐C_3_N_4_ can construct donor‐acceptor (D‐A) materials to enhance charge separation and light absorption for improving photocatalytic activity. Zhang et al. grafted a pair of spatially isolated donors (methoxyphenyl unit) and acceptors (anthraquinone unit) in g‐C_3_N_4_ edges [146]. By this, the photoinduced charge carriers directionally transfer to the two spatially separated dual active centers – e^−^ concentrated in the anthraquinone unit facilitate the 2e^−^ ORR, while the h^+^ in the methoxyphenyl unit enables efficient 4e^−^ WOR. Moreover, the anthraquinone unit with strong proton extraction capability also serves as a proton shuttle for accelerating the protonation of ORR intermediates to form *OOH and finally H_2_O_2_. As a result, the photocatalytic activity of this donor‐g‐C_3_N_4_‐acceptor photocatalyst surpasses traditional DP and PA catalysts, exhibiting a remarkable H_2_O_2_ yield of 6497.1 µM g^−1^ h^−1^ in pure water.

### 3.7 Other Strategies

**FIGURE 13:**
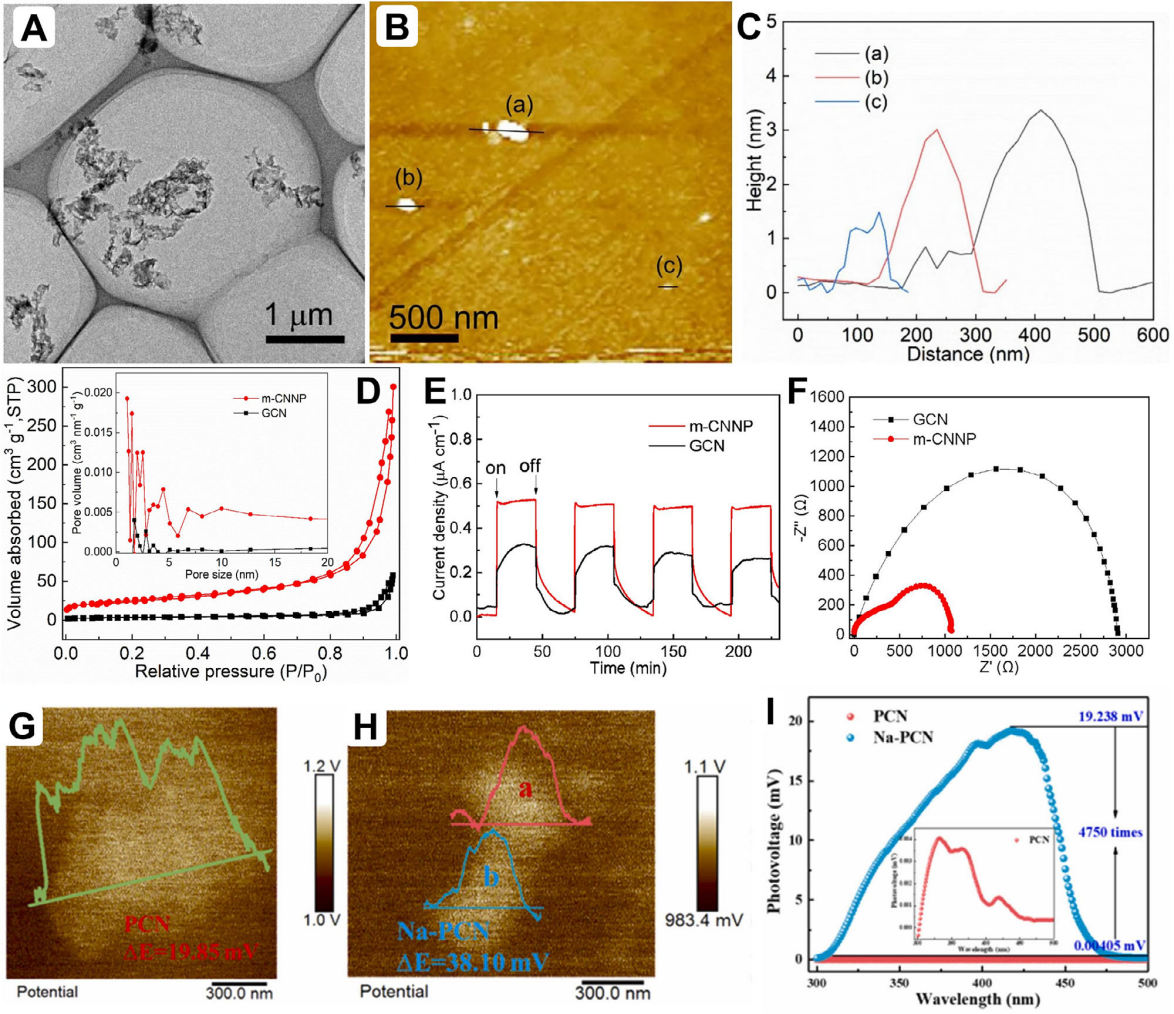
(A) TEM images of m‐CNNP. (B) Atomic force microscopy (AFM) image and (C) corresponding thickness data. (D) N_2_ adsorption/desorption isotherms of catalysts and pore size distribution. (E) Photocurrent responses. (F) EIS plots. Reproduced with permission [36]. Copyright 2021, Elsevier. (G) and (H) Kelvin probe force microscopy (KPFM) for determining the surface potentials. (I) Surface photovoltage (SPV) plots. Reproduced with permission [63]. Copyright 2024, Elsevier.

In addition to the aforementioned strategies, some other approaches have also been explored for promoting the photocatalytic H_2_O_2_ generation over g‐C_3_N_4_. For example, morphology tailoring is a helpful method in this context. The bulk g‐C_3_N_4_ is composed of numerous layers of nanosheets, leading to limited specific surface areas. The interlayer charge transfer is also unfavorable due to long‐distance transportation. Constructing nanostructures of g‐C_3_N_4_ by creating porous channels, aggrandizing the surface area and active sites, and altering the dimensions can significantly ameliorate the photocatalytic activity of g‐C_3_N_4_. Liu et al. prepared few‐layered g‐C_3_N_4_ nanoplates (m‐CNNP) with a thickness of 1–3 nm through co‐milling of pristine g‐C_3_N_4_ and glucose (Figure 13A–C) [36]. Compared with the bulk g‐C_3_N_4_, the 2D ultrathin morphology enables a significant increase in surface area and thus abundant reaction sites (Figure 13D), efficient charge separation and transfer (Figure 13E,F), as well as improved redox ability. Furthermore, the ultrathin structure can switch the pathway for H_2_O_2_ generation from the traditional two‐step single‐electron ORR to the one‐step two‐electron ORR. These ultrathin g‐C_3_N_4_ nanoplates show a H_2_O_2_ generation rate (43.07 µmol g^−1^ h^−1^) 4‐fold higher than that of bulk g‐C_3_N_4_. Li et al. reported an ultrathin acanthosphere‐like g‐C_3_N_4_ (ASCN) with N_V_ and carbonyl groups via shear stress‐assisted assembly [147]. The ultrathin nature of ASCN provides rich exposed active sites and improves charge carrier separation. Meanwhile, carbonyl groups concentrate holes to oxidize 4‐MBA, while N_V_ sites trap e^−^ for O_2_ reduction to H_2_O_2_. Accordingly, the ASCN catalyst shows significant photocatalytic activity for H_2_O_2_ production by coupling with 4‐MBA oxidation.

Increasing the crystallinity of g‐C_3_N_4_ effectively lessens internal and surface defects, which contribute to efficient charge separation and transfer and thereby enhance photocatalytic performance [148]. Jian et al. synthesized highly crystalline g‐C_3_N_4_ (Na‐PCN) by a sodium chloride‐assisted calcination method [63]. The presence of NaCl crystals enhances melem condensation and leads to strong interfacial confinement, facilitating the directed assembly of 2D nanosheets on the crystal plane. It was found that the high crystallinity contributes to a larger surface and zeta potential compared with pristine g‐C_3_N_4_ (PCN) (Figure 13G,H), indicating a strong built‐in electric field, thus promoting the exciton dissociation to form charge carriers (Figure 13I). Moreover, the formation of nanocrystals induces the rapid charge migration by shortening the corresponding distance. Together with the advantage of abundant surface −C≡N/−OH groups for O_2_ adsorption, the H_2_O_2_ photosynthesis over Na‐PCN reaches a remarkable rate of 16.01 mmol g^−1^ h^−1^) using ethanol as the sacrificial agent, 77‐fold of PCN.

**FIGURE 14:**
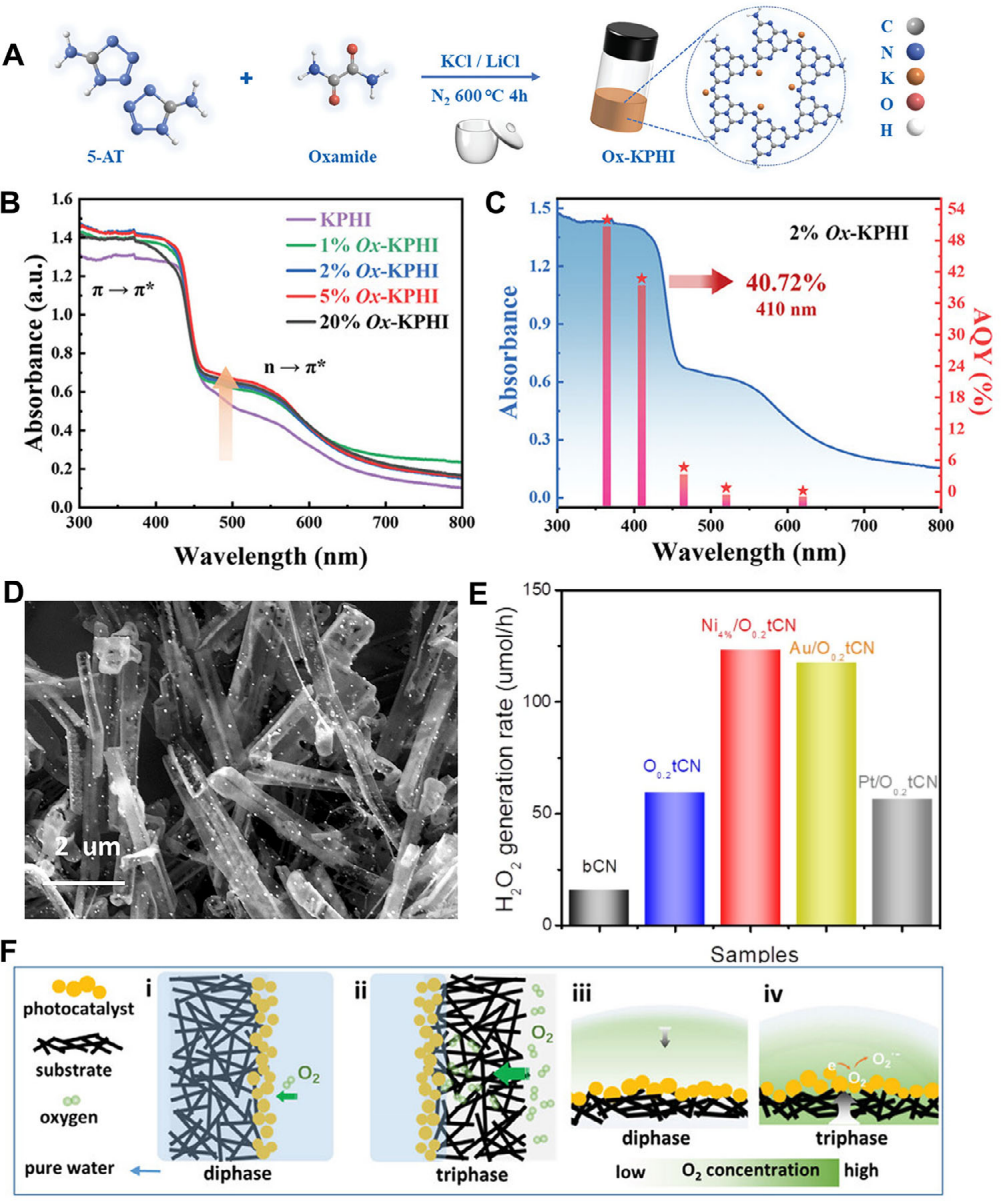
(A) Schematic illustration of the synthesis process of x%Ox‐KPHI. (B) UV–vis diffuse reflectance spectra (DRS) of pristine KPHI and oxamide‐modified KPHI samples. (C) Wavelength‐dependent UV–vis absorption spectrum and AQY for 2%Ox‐KPHI. Reproduced with permission [151]. Copyright 2024, Wiley. (D) HAADF‐STEM image of Ni/OtCN catalyst, with Ni nanoparticles highlighted as bright spots. (E) Photocatalytic H_2_O_2_ production rates over bCN, O_0.2_tCN, and M_4%_/O_0.2_tCN (M = Ni, Au, Pt) catalysts [155]. Copyright 2022, Elsevier. (F) Comparison of diphase and triphase photocatalytic systems: (i) diphase (O_2_‐saturated water), (ii) triphase configurations, and local O_2_ concentration profiles for (iii) diphase vs. (iv) triphase systems. Reproduced with permission [44]. Copyright 2021, Wiley.

As a type of crystalline carbon nitride material, PHI has recently emerged for efficient photocatalytic H_2_O_2_ production due to its well‐defined ionic framework and superior electronic properties [27, 149]. Unlike conventional g‐C_3_N_4_, PHI exhibits an ordered structure with alkali metal intercalation (e.g., K^+^ or Na^+^), which enhances charge carrier mobility and separation efficiency [66, 150]. The material's extended π‐conjugation and crystallinity further contribute to improved photostability and reduced charge recombination compared to amorphous carbon nitrides. Recent studies have demonstrated that PHI achieves high H_2_O_2_ production rates (46.8 mmol g^−1^ h^−^^1^ under visible light) and excellent AQY (up to 41% at 410 nm) in the absence of sacrificial agents [65, 151]. Moreover, through the abovementioned modifications such as defect engineering [151], heterostructure construction [152], and single‐atom doping [153], the photocatalytic activity of PHI can be further improved. For example, Tong et al. simultaneously introduced structural distortions and defect sites (–C≡N groups and N_V_) into the K‐PHI framework (Figure 14A), which enables an apparent quantum yield of 41% at 410 nm for H_2_O_2_ generation by means of *n* → π* electronic transition activation (Figure 14B,C) [151]. Teng et al. reported K‐PHI loaded with low‐valent Au SAs. The strong K–N bonds in the PHI matrix stabilized Au near a 0 oxidation state, modifying the band structure to trap localized h^+^. These h^+^ drove 1e^−^ water oxidation to form •OH while dissociating H from H_2_O, significantly boosting O_2_ reduction to H_2_O_2_ [153, 154].

In spite of multiple approaches for improving the photocatalytic performance of g‐C_3_N_4_ by different mechanisms, it's difficult for a single modification to simultaneously optimize different photocatalytic properties of g‐C_3_N_4_. Therefore, multiple strategies are sometimes employed to optimize the materials from different aspects. For example, Du et al. prepared oxygen‐doped tubular g‐C_3_N_4_ with nickel nanoparticle loading for the photosynthesis of H_2_O_2_ (Figure 14D) [155]. This material adopts the advantages of various modifications, including morphology design, heteroatom doping, and metal junction engineering. The hollow structure attaches a large surface area for providing abundant reactive sites and promoting visible light absorption; the oxygen doping and Ni loading accelerate the charge separation and 2e^−^ oxygen reduction for H_2_O_2_ generation (Figure 14E). Ba et al. reported g‐C_3_N_4_ ultrathin nanosheets engineered with both N_V_ and cyano groups [53]. Through a combination of morphology design and defect engineering, the modified g‐C_3_N_4_ exhibits strengthened light adsorption, promoted charge separation/transfer efficiency, and rich surface active sites, which work together to maximize the H_2_O_2_ synthesis efficiency.

Development of efficient g‐C_3_N_4_‐based photocatalysts for H_2_O_2_ production requires a fundamental understanding of structure‐activity relationships and reaction mechanisms, which can be achieved through the synergistic application of in situ characterizations and theoretical calculations. In situ techniques provide unprecedented insights into dynamic processes under actual photocatalytic conditions. Time‐resolved spectroscopic technologies like transient absorption spectroscopy and operando PL reveal charge carrier lifetimes and recombination pathways [156, 157], enabling the rational design of strategies to enhance charge separation. Surface‐sensitive techniques, such as in situ FTIR and Raman spectroscopy, identify critical reaction intermediates (e.g., *OOH species) and active sites [158], while in situ XPS tracks chemical state evolution of the catalyst surface during operation [102]. DFT calculations predict electronic structure modifications through doping (e.g., S, P, or metal atoms) and defect engineering (e.g., N_V_), revealing how these alterations affect band structure, charge localization, and adsorption energetics of key species (e.g., O_2_) [159, 160]. Advanced molecular dynamics simulations model the interaction between reactants and catalyst surfaces under realistic conditions, helping identify rate‐limiting steps in the complex H_2_O_2_ formation pathway [151]. The integration of these approaches has led to breakthroughs in understanding the delicate balance between 2e^−^ oxygen reduction and competing 4e^−^ pathways, as well as strategies to suppress H_2_O_2_ decomposition. This combined experimental–theoretical framework is essential for designing g‐C_3_N_4_ photocatalysts that simultaneously achieve high H_2_O_2_ yield, excellent stability, and solar‐to‐chemical efficiency, paving the way for practical applications in clean energy and environmental remediation.

Sacrificial agents such as various kinds of alcohols serve as essential components in g‐C_3_N_4_‐based photocatalytic systems for H_2_O_2_ generation, functioning through three primary mechanisms that collectively enhance both the efficiency and selectivity of the process. Firstly, these agents dramatically improve charge carrier separation efficiency by preferentially consuming photogenerated h^+^, which addresses the rapid charge recombination issue for g‐C_3_N_4_. With the VB h^+^ consumed by sacrificial agents, the lifetime of CB e^−^ can be significantly extended, increasing the population of e^−^ available for ORR. Secondly, sacrificial agents can directly interact with the catalyst or reactants such as O_2_ to facilitate H_2_O_2_ formation [161, 162]. Xu et al. reported that the carboxylic groups in EDTA could interact with O_2_ by forming an H‐bond, leading to enhanced O_2_ dissolution in water [162]. Importantly, such interaction can facilitate the O_2_ reduction in thermodynamics and kinetics and protect the generated H_2_O_2_ from oxidative decomposition. Thirdly, certain sacrificial agents enable entirely novel H_2_O_2_ generation pathways that circumvent traditional ORR limitations. Furfuryl alcohol (FFA) can selectively react with singlet oxygen (^1^O_2_) to produce H_2_O_2_. Xu et al. applied this reaction over an excellent amidated‐C_3_N_4_ photocatalyst that could efficiently and selectively activate O_2_ to ^1^O_2_, achieving efficient production of H_2_O_2_ at a rate of 14.6 mmol g_cat_^−1^ h^−1^ and an AQY of 44.1% at 420 nm [68].

Reactor optimization is another direction for enhancing photocatalytic H_2_O_2_ production. A conventional suspension batch reaction system is faced with limitations such as low solubility of O_2_ in water and insufficient illumination, which strongly limit the H_2_O_2_ production performance. Hence, recent advances have focused on the construction of novel reaction systems. Gas‐diffusion systems, employing porous electrodes or membranes (e.g., carbon‐based gas‐diffusion electrodes), enhance O_2_ supply by facilitating efficient tri‐phase (gas–liquid–solid) contact, significantly boosting H_2_O_2_ yields (Figure 14F) [44]. Droplet‐based reaction systems leverage microdroplets or aerosolized interfaces to maximize light penetration and O_2_ diffusion, achieving ultrahigh local concentrations [163, 164]. Continuous microbatch photoreactors combine flow chemistry with photocatalysis, enabling precise control over residence time and improved mass transfer [165]. Meanwhile, floating photocatalyst systems immobilize catalysts at the air–water interface, minimizing light scattering and ensuring direct O_2_ access [166, 167]. These innovative designs address critical challenges in scalability, efficiency, and sustainability for solar‐driven H_2_O_2_ synthesis.

## 4 Conclusion and Outlook

Artificial photosynthesis of H_2_O_2_ by g‐C_3_N_4_‐based materials is a topic of great interest. A series of novel g‐C_3_N_4_‐based photocatalysts have been recently developed to elevate H_2_O_2_ synthesis efficiency. In this review, we introduced the fundamental principles of photocatalytic H_2_O_2_ synthesis and systematically summarized the modification strategies of g‐C_3_N_4_ for boosting the performance on H_2_O_2_ generation. Representative advanced g‐C_3_N_4_‐based photocatalysts were discussed in detail to provide a more comprehensive understanding of the rules for designing g‐C_3_N_4_‐based materials. With the significant progress that has been achieved recently, the H_2_O_2_ synthesis over g‐C_3_N_4_‐based photocatalysts unleashes more and more possibilities toward practical applications.

To realize practical H_2_O_2_ photosynthesis, there are still quite a few challenges to be addressed. First of all, the efficiency of H_2_O_2_ synthesis remains low, as reflected by the Φ_AQY_ values falling significantly below the theoretical maximum. On the intrinsic side, the bulk and surface recombination of photogenerated charge carriers remains a primary pathway for efficiency loss, while the kinetic barriers of the surface catalytic reactions may also restrict the utilization of these charges. Extrinsically, the current reactor configuration may suffer from inefficient mass transport of reactants/products and suboptimal utilization of the incident light. For future improvements, near‐term efforts should prioritize electronic‐structure engineering to enhance charge separation and migration. Once the intrinsic material activity is sufficiently improved, advanced reactor design will become decisive for practical, large‐scale applications by optimizing flow and illumination fields.

Secondly, the overall photosynthesis of H_2_O_2_ from H_2_O and O_2_ holds great promise for the in situ generation and application of H_2_O_2_ without sacrificial agents. However, this approach remains highly challenging, primarily due to the sluggish kinetics of the WOR and the limited supply of protons in neutral or alkaline aqueous environments, which hinders the efficient ORR. Research efforts should focus on designing catalytic systems with synergistic active sites to respectively accelerate the WOR and ORR kinetics, considering the reaction microenvironment. Thirdly, from a practical application perspective, current photocatalytic systems face significant hurdles in terms of catalyst stability and scalability. The materials often suffer from photo‐corrosion, structural degradation, and a gradual loss of active sites under prolonged illumination. Moreover, scaling up the process while maintaining the high efficiency demonstrated in laboratory settings remains a formidable challenge. Future work needs to address these issues at both the material and system levels.

In this context, it is of great significance to further develop advanced strategies capable of addressing these issues so as to realize practical solar‐to‐chemical conversion by g‐C_3_N_4_‐based synthesis of H_2_O_2_. Future works focusing on the following aspects may be very valuable.

1. Improving the absorption of visible or even infrared light is the basis of efficient solar‐to‐chemical conversion. To further improve the light absorption of g‐C_3_N_4_, innovative synthetic strategies should focus on atomic‐level modifications and advanced heterostructure engineering – developing vapor‐phase atomic layer doping techniques to achieve uniform heteroatom incorporation while preserving the polymeric framework, optimizing molten‐salt‐assisted synthesis to create controlled defects with minimal structural damage, and designing core–shell architectures with atomic layer–deposited metal oxides. Furthermore, emerging techniques like atomic layer deposition for precise interfacial control or machine learning–guided synthesis for optimal material combinations represent promising avenues for developing future g‐C_3_N_4_‐based photocatalysts with superior light absorption.
2. The charge separation efficiency strongly determines the kinetics of ORR and H_2_O_2_ generation. As mentioned above, almost all modification technologies pay much attention to the charge separation process. Construction of g‐C_3_N_4_‐based hetero‐/homo‐junctions is a very effective approach for this purpose due to the spatial separation of photogenerated charge carriers. Especially, coupling modified g‐C_3_N_4_ with other photocatalysts into heterojunctions could simultaneously take advantage of the heterojunction and other modifications, such as defect creation and heteroatom doping. Thereby, maximum optimization of charge separation efficiency can be realized.
3. Coupling specific oxidation reactions with ORR is an advanced strategy of killing two birds with one stone. As the ORR for H_2_O_2_ generation only consumes the photoinduced e^−^, the contemporaneously generated h^+^ is usually consumed for water oxidation, or it will recombine with e^−^. Due to the sluggish kinetics for water oxidation over g‐C_3_N_4_, the ORR could be inhibited accordingly. If coupled with some faster oxidative conversion reactions (e.g., biomass to valuable small organics), the ORR shall be enhanced, and a higher H_2_O_2_ yield can be obtained. Meanwhile, the h^+^‐induced oxidation products can also be collected. This could be a promising photocatalytic system simultaneously utilizing e^−^ and h^+^ for utmost economic benefit.
4. The reaction conditions for ORR need careful design for maximizing the H_2_O_2_ synthesis. There are various parameters that could essentially affect the ORR kinetics, such as solution pH, ion strength, catalysts’ dosage, irradiation spectra and intensities, temperature, etc. Though some reports have shown the critical effect of these parameters for H_2_O_2_ generation, a thorough study of the specific effects is still rare until now. Paralleling with material design, understanding and optimizing the reaction conditions can help to obtain further improved catalytic performance.
5. Overall, H_2_O_2_ photosynthesis is ideal for in situ applications such as water purification, disinfection, and chemical synthesis. In view of the key challenges in efficiency, balancing the WOR and H_2_O_2_ decomposition is the critical issue. The WOR can be enhanced by rational catalyst design to improve the hole oxidation potential and selectivity for adsorption and activation of H_2_O, enabling higher thermodynamics and kinetics of WOR to generate O_2_, or even directly H_2_O_2_ to realize dual H_2_O_2_ production. Furthermore, constructing hybrid systems (e.g., integrating photocatalysis with electro‐, piezo‐, or biocatalysis) can facilitate efficient charge separation and spatially isolate WOR and H_2_O_2_ generation sites, simultaneously accelerating both reactions while minimizing H_2_O_2_ degradation.
6. The in situ applications of H_2_O_2_ provide easy access for addressing the energy and environmental issues. Considering the currently limited yield of H_2_O_2_, it would be better to use it directly without further separation or transportation. The in situ activation of H_2_O_2_ into hydroxyl radicals is critical for environmental treatment. In this regard, the selective activation of H_2_O_2_ needs to be taken into consideration for the catalyst design. In addition, considering the complex compositions in specific water, the photocatalysts need to have a strong anti‐jamming capability to maintain the activity for H_2_O_2_ synthesis.
7. The integration of in situ characterizations and theoretical calculations holds tremendous potential for advancing g‐C_3_N_4_‐based photocatalytic H_2_O_2_ production. Future developments will likely focus on higher spatiotemporal resolution techniques, such as ultrafast in situ spectroscopy and environmental TEM, to capture transient intermediates and surface dynamics at atomic scales. Coupled with multiscale theoretical modeling, these approaches will unravel the complex interplay between charge transfer, surface reactions, and catalyst degradation under operational conditions. Machine learning‐assisted high‐throughput screening will accelerate the discovery of optimal dopants, defects, and heterostructures, while advanced simulations incorporating solvent effects and interfacial phenomena will provide more realistic performance predictions. The ultimate goal is to establish design rules for g‐C_3_N_4_ catalysts that simultaneously maximize H_2_O_2_ yield, selectivity, and stability.
8. The path to practical application of g‐C_3_N_4_ depends on overcoming key hurdles in scalability and operational stability. To this end, improving the robustness of g‐C_3_N_4_‐based systems should be a primary focus. Research needs to move beyond merely enhancing charge separation and tackle the issue of material degradation head‐on, for instance, by developing more crystalline phases or composite architectures with inherently stable properties. Equally important is establishing synthesis routes for these materials that are both scalable and cost‐effective. At the system level, validation must progress from idealized lab settings to outdoor environments, where performance under real‐world variations in sunlight, temperature, and humidity can be assessed. Ultimately, bridging the gap from lab‐scale innovation to industrial‐scale solar H_2_O_2_ production will require a coordinated strategy that tightly integrates advanced material design with practical reactor engineering.

## Conflicts of Interest

The authors declare no conflicts of interest.

## Data Availability

The data that support the findings of this study are available from the corresponding author upon reasonable request.
